# Spatio-temporal patterns in juvenile habitat for 13 groundfishes in the California Current Ecosystem

**DOI:** 10.1371/journal.pone.0237996

**Published:** 2020-08-21

**Authors:** Nick Tolimieri, John Wallace, Melissa Haltuch

**Affiliations:** 1 Conservation Biology Division, Northwest Fisheries Science Center, National Marine Fisheries Service, National Oceanic and Atmospheric Administration, Seattle, WA, United States of America; 2 Fisheries Research, Analysis and Monitoring Division, Northwest Fisheries Science Center, National Marine Fisheries Service, National Oceanic and Atmospheric Administration, Seattle, WA, United States of America; Hellenic Centre for Marine Research, GREECE

## Abstract

Identifying juvenile habitats is critical for understanding a species’ ecology and for focusing spatial fishery management by defining references like essential fish habitat (EFH). Here, we used vector autoregressive spatio-temporal models (VAST) to delineate spatial and temporal patterns in juvenile density for 13 commercially important species of groundfishes off the US west coast. In particular, we identified hotspots with high juvenile density. Three qualitative patterns of distribution and abundance emerged. First, Dover sole *Microstomus pacificus*, Pacific grenadier *Coryphaenoides acrolepis*, shortspine thornyhead *Sebastolobus alascanus*, and splitnose rockfish *Sebastes diploproa* had distinct, spatially-limited hotspots that were spatially consistent through time. Next, Pacific hake *Merluccius productus* and darkblotched rockfish *Sebastes crameri* had distinct, spatially limited hotspots, but the location of these hotspots varied through time. Finally, arrowtooth flounder *Atheresthes stomias*, English sole *Parophrys vetulus*, sablefish *Anoplopoma fimbria*, Pacific grenadier *Coryphaenoides acrolepis*, lingcod *Ophiodon elongatus*, longspine thornyhead *Sebastolobus altivelis*, petrale sole *Eopsetta jordani*, and Pacific sanddab *Citharichthys sordidus* had large hotspots that spanned a broad latitudinal range. These habitats represent potential, if not likely, nursery areas, the location of which will inform spatial management.

## Introduction

The spatial distribution of a species is one of the fundamental aspects of its ecology [1, 2] and is a component of many conservation and management plans [2–5]. For marine fishes, understanding a species’ spatial distribution is necessary for delineating Essential Fish Habitat (EFH), which is important for an ecosystem-based approach to fisheries management, and is part of the Magnussen-Stevens Act (as amended in 1996). In the United States, the National Marine Fisheries Service (NMFS) and regional fisheries councils are required to identify EFH [6, 7]. However, broadly defined as “those waters and substrate necessary to fish for spawning, breeding, feeding and/or growth to maturity,” EFH quickly comes to include most waters within the exclusive economic zone (https://www.fisheries.noaa.gov/resource/map/essential-fish-habitat-mapper) when EFH is summed across species, making the prioritization of conservation and management actions difficult [8, 9].

While a species may occupy many habitats or areas, some areas are likely to be more ecologically important than others to the long-term persistence and productivity of the species. For example, protecting core adult habitat for adult rockfishes *Sebastes* spp. has been shown to lead to increases in reproductive output for these populations [10]. Alternatively, restoration of juvenile habitats for red drum *Sciaenops ocellatus* has been shown produce larger increases in population growth rate and adult abundance than would protection and restoration of adult habitat [8]. These ‘essential’ EFHs [8] are termed Habitat Areas of Particular Concern (HAPC) due to their increased importance for the species’ population dynamics [4, 5]. Locating areas that support high densities of juvenile fishes is a first step towards understanding EFH and HAPC and being able to apply spatial management plans [11, 12]. Nursery areas are juvenile habitats that contribute disproportionately more individuals to the adult population than average (usually defined in terms of production per unit area) due to higher juvenile: (1) density, (2) growth rate, (3) survival, and (4) movement to adult habitat [13]. Here, we focus on the first criteria: density of juvenile fishes.

Strong recruitment events can determine age structure and stock size in marine fishes [14, 15], suggesting that the location, quality, and availability of juvenile habitat are essential to the health of these populations [8, 16–19]. For example, recruitment to five populations of sole *Solea solea* in the northeast Atlantic is related to the area of suitable juvenile habitat [19], and the abundance of age-0 cod *Gadus morhua* is correlated with the availability of eelgrass habitat [12]. While there has been a substantial focus on nearshore and estuarine habitats [8, 16, 20–22], less work has gone into quantifying the spatial distribution of juvenile habitat for deeper marine species across large spatial scales.

Starting with an areal delineation of a species juvenile habitat is useful because bottom type alone does not necessarily define high quality areas. Other factors, such as larval supply, food availability, and connectivity with adult habitats can be important. For example, there are substantial seagrass beds that appear to be suitable habitat for juvenile queen conch (*Stombas gigas*) in the Bahamas [23]. However, settlement occurs to specific areas where tidal circulation concentrates larvae, and conch growth is highest in areas where macroalgal production exceeds surrounding areas. In other cases, nursery habitat can be ephemeral and temporally variable when species recruit to biogenic habitats [11, 12, 24, 25] or respond to changes in oceanographic or climate drivers like temperature [26, 27]. In fact, shifts in juvenile distributions related to climate or other factors may hamper management efforts [26].

**Fig 1:**
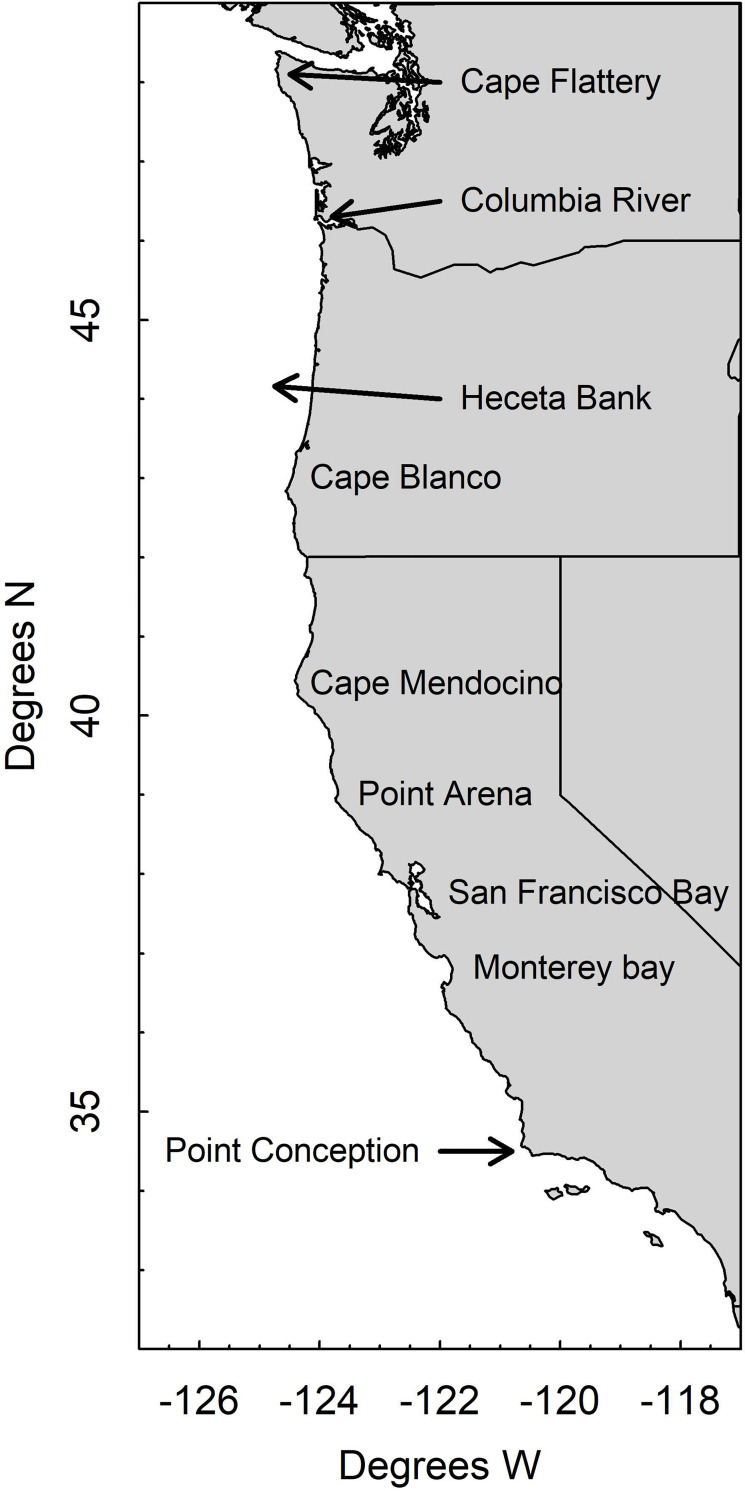
West coast of the United States showing geographic features referenced in the text. Bathymetric lines are 200, 600 and 1200-m isobaths.

Quantifying the spatial distribution and abundance of juvenile fishes allows us to identify juvenile habitats and potential nursery areas as well as to begin developing hypotheses concerning the factors controlling the spatial distribution of juvenile fishes (e.g., current regimes that may entrain larvae). Here, we used data from the Northwest Fisheries Science Center’s US West Coast Groundfish Bottom Trawl Survey [WCGBTS, 28] to quantify the spatial distribution of juvenile fishes for 13 species (five flatfishes and eight roundfishes, Table 1) in waters off the west coast of the United States (Fig 1). We used vector-autoregressive spatio-temporal modeling [VAST, 2, 29, 30] to estimate species distribution models and quantify spatial and temporal patterns in juvenile density and identify juvenile habitat (in terms of spatial distributions) for each species. We ask: (1) are there juvenile hotspots along the coast for each of the species or are juveniles distributed homogeneously? (2) Is there spatial fidelity over time in juvenile hotspots for each of the species? And (3) are spatial patterns of juvenile biomass stable through time? Finally, (4) we also estimate an annual, coast-wide index of juvenile biomass for each species, the species effective area occupied, and its center of gravity (CoG).

**Table 1:** List of species included in the analyses.

Common name	Species	Family	Maximum length (cm)	VAST Age classes	VAST Depth (m)	Trawls per year
Arrowtooth flounder^a^	*Atheresthes stomias*	Pleuronectidae	22	1	50–470	234–326
Dover sole	*Microstomus pacificus*	Pleuronectidae	17	1, 2	50–465	285–439
English sole^a^	*Parophrys vetulus*	Pleuronectidae	16	1	50–140	91–182
Pacific sanddab	*Citharichthys sordidus*	Paralichthyidae	13	0, 1	50–245	230–342
Petrale sole	*Eopsetta jordani*	Pleuronectidae	21	1, 2	50–200	199–300
Lingcod^a^	*Ophiodon elongatus*	Hexagrammidae	25	0	50–240	180–308
Pacific hake	*Merluccius productus*	Merlucciidae	15	0–1	50–700	369–568
Pacific grenadier	*Coryphaenoides acrolepis*	Macrouridae	3	ca. 1^b^	490–1275	131–219
Sablefish*	*Anoplopoma fimbria*	Anoplopomatidae	29	0	50–475	310–437
Darkblotched rockfish	*Sebastes crameri*	Sebastidae	15	0,1	80–240	147–222
Longspine thornyhead	*Sebastolobus altivelis*	Sebastidae	7	< 5^b^	385–1245	172–302
Shortspine thornyhead	*Sebastolobus alascanus*	Sebastidae	8	< 5^b^	160–625	160–258
Splitnose rockfish	*Sebastes diploproa*	Sebastidae	10	0, 1	65–460	298–457

Maximum length (cm) is the maximum size of individuals used in the analysis and is the maximum length of the oldest age class based on length-at-age distributions of aged fishes. Age is the estimated age classes included in VAST modeling. Species without age classes specified do not have reliable age data, therefore only size was used. Depth (m) is the range of depths for the positive observations.

^a^Species with single juvenile age class (either age-0 or age-1)—resulting pattern and index considered an index of annual recruitment. Species without age-class designations did not have otolith-derived ages from the trawl survey.

^b^ Trawl survey data did not contain ageing information for these species. Age of fishes included in the analysis were estimated from the literature for these species: [Pacific grenadier: 31, longspine thornyhead: 32, shortspine thornyhead: 33].

## Materials and methods

### Data source

We used data for 2003–2018 from the Northwest Fisheries Science Center’s (NWFSC) U.S. West Coast Bottom Trawl Survey of Groundfish Resources off Washington, Oregon, and California [WCGBTS, 28]. The survey is takes depth-stratified, random samples that span approximately 32–48.58°N and 55–1280 m and is conducted in two passes from May to October with over 600 trawls per year [see 28, 34 for a detailed description of the sampling design]. Survey vessels start at Newport, Oregon and initially head north to Cape Flattery before heading south to San Diego [34]. The survey uses a standard Aberdeen-net with 25.9-m headrope, 31.7-m footrope, and an additional 3.8-cm liner extending from the middle of the net through the codend, to retain smaller fish and invertebrates. The net was towed at ~2.2 knots for a nominal 15 minutes [an average of 20 minutes on bottom time including lift-off lag, 35] and swept area ranged from 0.8 to 4.5 ha (median: 1.7). We included only those hauls deemed acceptable for stock assessment.

### Data selection and processing

Data selection and processing involved two issues. First, ideally we would have analyzed only age-0 or the youngest observed age class fish to provide estimates of recruitment distributions and abundance. However, the survey trawls often do not catch small juvenile fish in high numbers, resulting in a large number of tows with zero catch for the smallest age classes. For some species, we had to include multiple age classes in the analysis (sum biomass across age classes) in order to achieve a positive-definite Hessian and assure that the VAST model converged (see *Statistical analyses* below, Table 1). Thus, for some species (lingcod, sablefish, arrowtooth flounder, and English sole), the analysis examined a single juvenile age class (either age-0 or age-1) and the resulting pattern and index can be considered an index of annual recruitment. However, for the remainder of the species, the analysis quantifies juvenile density because we had to combine several juvenile age classes. Hereafter, we refer to both cases as juvenile density.

Second, while the survey data include subsamples of individual fish lengths, weights, and age structures–with ages determined from otoliths (except for Lingcod for which dorsal fin rays are used)–there are always more length measurements than ages due to limited processing capacity. Since the size distributions for a multiple ages can overlap, we used length-at-age relationships from the aged individuals to set a maximum length for inclusion in a given age class for those individuals that were not aged. We then pooled all individuals of this maximum length or below for the statistical analyses, which do not distinguish age classes within the statistical model.

To set the maximum length for each species, we examined the length distributions of aged individuals by 1-cm bins. The longest length bin where younger individuals outnumbered older individuals was set as the maximum length for the younger age class. For example, the length distribution for age-0 and age-1 sablefish overlapped between 26 and 31cm (Table A in S1 Appendix). However, 29 cm is longest length bin where there were more age-0 fish (ten) than age-1 fish (nine). At 30 cm there were more age-1 than age-0 fish. Therefore we set a maximum length of 29 cm for inclusion of unaged sablefish in the age-0 group.

We then extrapolated the full tow weight for the lengths selected based on their weighted proportion in that species’ subsample that was measured for length. If the extrapolated tow-level species weights from the initial multispecies sample were already in the database, they were used as given. The length-weight relationship for each species was calculated from the available data and used when a weight was missing for an individual fish. We also used the length-weight relationships to check for weights that were outliers, for example, in cases where a basket holding the specimen was not correctly tared. In such cases, the calculated weight was used instead. The implied assumption that the length is the correct measurement and not the weight. This assumption is justified given the ease of taking length measurements using the digital length boards employed by the survey. The trawl survey data did not contain age-structure information for Pacific grenadier and the two thornyhead species. Instead, we used age-at-length information from Andrews, Cailliet [31; Pacific grenadier], Taylor and Stephens [33, shortspine thornyhead], and Stephens and Taylor [32, longspine thornyhead] as a guide. For each species, lengths (in 1 cm intervals) were added to the analysis until we obtained acceptable model fits, and based on the published information, we estimated the maximum age of individuals in the analysis.

### Statistical analyses

We used vector-autoregressive spatio-temporal modeling [R package VAST, version 4.1, 2, 29, 30] to quantify the spatial and temporal trends of juvenile biomass density (hereafter, density for brevity) for each of the 13 species. VAST is a species distribution modeling approach that effectively reproduce species distributions and provide good estimates of abundance and associated error [36]. VAST performs well with linear covariates, but has limited ability to include non-linear relationships beyond general quadratic ones compared to generalized additive models or branched regression trees [36]. We use VAST here because we are primarily interested in estimating species distributions and an annual index of abundance.

Catch per unit effort (CPUE) was the dependent variable, calculated as the extrapolated abundance estimate of juveniles divided by the swept area of the net. The year of capture, pass, vessel, tow location (latitude and longitude), and depth were the predictor variables. The trawl survey is done in two passes between May and October. Pass therefore accounts for intra-annual variation the time of sampling, and the inclusion of pass is consistent with VAST models used to produce biomass indices for west coast stock assessments. Since the goal was to model distributions and not test for explanatory variables, we did not include additional environmental predictors in the models. We applied one common intercept across years, which allowed spatial variation to be explained by spatial and spatio-temporal variation terms, both of which were included in the model. This parameterization prevents the model from forcing biomass to increase or decrease coast-wide in a given year (thereby potentially overestimating recruitment in some areas) as would be the case for yearly intercept. We used gamma-distributed errors for the positive catch rates with a Poisson-link function. This approach approximates a Tweedie distribution, which has positive mass at zero but is otherwise continuous, but is more computationally efficient [30, 37]. Previous work has shown that this approach generally provides a better fit to the WCGBTS data than does a conventional delta model [30, 38]. The extrapolation grid or stratum area was defined based on the data extent for each species separately (Region = ‘Other’ in VAST, Table B in S1 Appendix). Trawls with zero catch for the target species and within the stratum area were included in the analysis. We used 600 knots [locations at which spatial processes are estimated by VAST, used for computational efficiency; 30] for all model runs. See Thorson [30] for more detail on VAST and Table B in S1 Appendix for more detail on the VAST parameterization used here.

### Hotspots

We arbitrarily defined juvenile hotspots as areas where estimated juvenile density (biomass) derived from VAST was in the top 20% of its maximum density (80%+) over the study period (red areas the following distribution figures). We define secondary hotspots as areas where juvenile density was in the top third (66% +) of its maximum density over the study period (orange areas in the following distribution figures). Additionally, we examined hotspots at two temporal scales: within years and across years (‘All Years’ on the following distribution figures). All Years was the average juvenile density from 2003–2018. Therefore, areas that consistently had moderate juvenile density may appear as a hotspots across all years, though they may not necessarily qualify as a hotspot at an annual scale.

## Results

We observed three general, qualitative patterns of juvenile density (biomass) along the West Coast: (1) species with distinct, spatially-limited hotspots that were consistent through time; (2) species with distinct, spatially-limited hotspots that spatially varied through time; and (3) species with large regional hotspots that spanned a broad latitudinal range but were depth limited. Additionally, we estimated the time series of juvenile density for each species, which illustrated that the prevalence and intensity of juvenile hotspots was often related to density. Finally, we also report the effective area occupied and CoG for each species.

**Fig 2:**
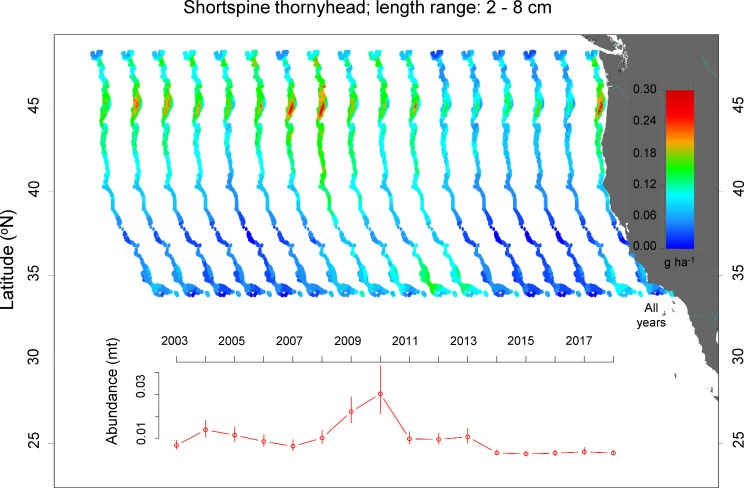
Spatial distribution and annual abundance index of juvenile shortspine thornyheads. All Years was the average juvenile density from 2003–2018.

Dover sole, English sole, Pacific grenadier, shortspine thornyhead, and splitnose rockfish had distinct and spatially limited hotspots of juvenile density (shortspine thornyhead, Fig 2; others Figs A-E in S1 Appendix; see S2 Appendix for diagnostic plots including QQ-plots and plots of the spatial residuals for the presence/absence portion of the model). For example, the density of juvenile shortspine thornyheads was fairly consistent through time but peaked in 2009–2010, after which it declined (Fig 2). There was a single, distinct across-years hotspot at approximately 45° N in waters between 160–625 m, just north of an area with an expansive area of shallower shelf habitat that includes Heceta, Perpetua, and Stonewall Banks (Fig F in S1 Appendix). The hotspot north of the banks had primary or secondary hotspots in most years until approximately 2014, after which juvenile density was very low, but it was most obvious in 2010 when juvenile density was highest. There was a small, secondary, across-years hotspot adjacent to the Columbia River mouth, with a primary hotspot at this location in 2010 when the density of shortspine juveniles peaked.

**Fig 3:**
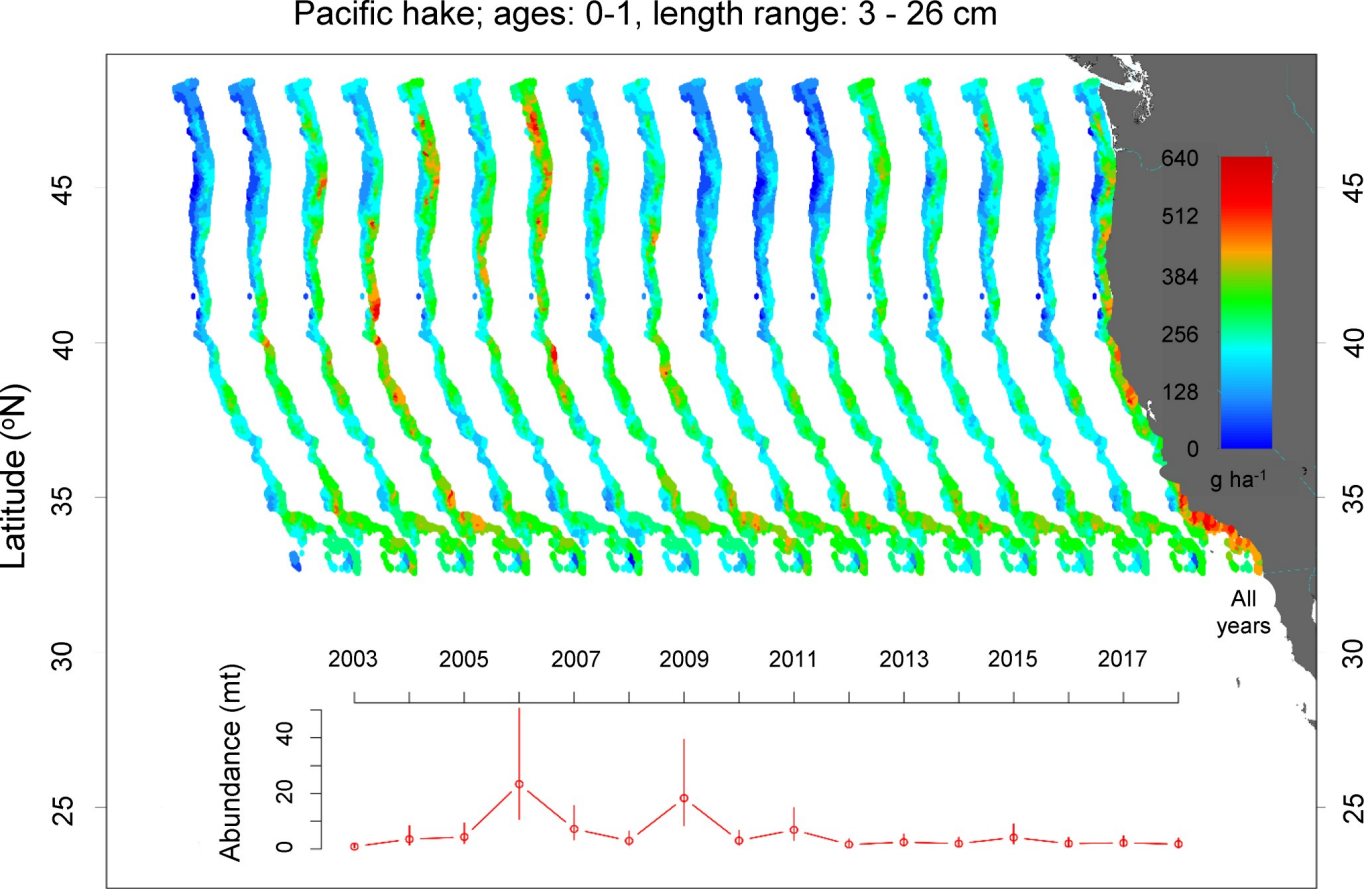
Spatial distribution and annual abundance index of juvenile Pacific hake. All Years was the average juvenile density from 2003–2018.

Pacific hake (Fig 3) and darkblotched rockfish (Fig G in S1 Appendix) also showed distinct hotspots of juvenile density, but there was more temporal variation in the location of these hotspots. For example, Pacific hake showed peaks in juvenile density in 2006 and 2009. Juveniles (age-0 and age-1) were caught between 50–700 m. Within a given year, they showed localized areas of high density, but the location of these hotspots varied from year to year. While the location of the hotspots did not appear related to the density of juveniles, hotspots were most apparent in 2006 and 2009 when the density of juvenile hake showed peaks. For example, in 2006 hotspots occurred north and south of Cape Blanco and around Point Conception. However, in 2009 there was a hotspot to the north on either side of the Columbia River outflow. Juvenile density was consistently moderate south of Cape Mendocino resulting in across-years hotspots north of San Francisco Bay and around Point Conception (‘All Years’, Fig 3).

**Fig 4:**
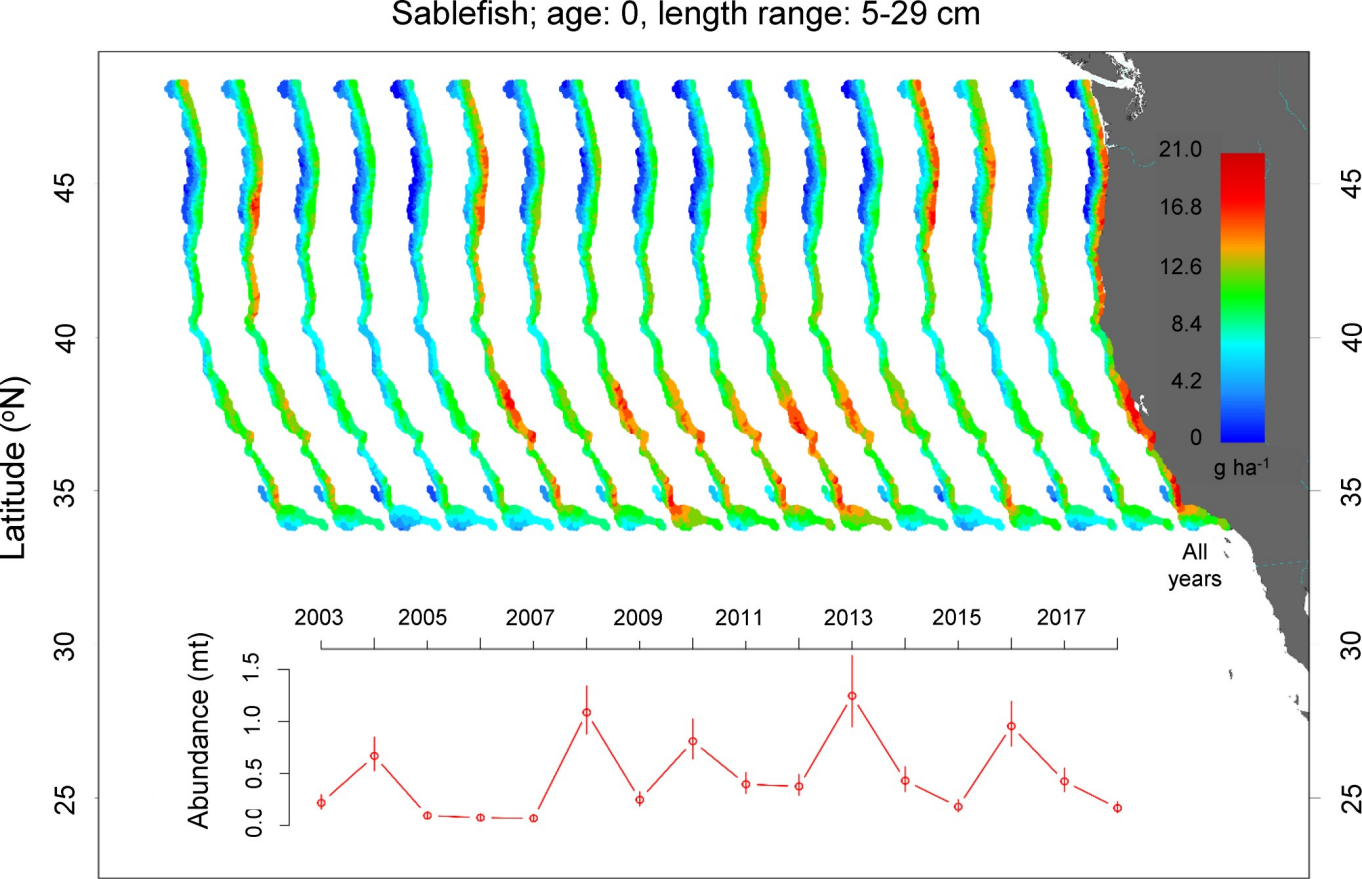
Spatial distribution and annual abundance index of juvenile sablefish. All Years was the average juvenile density from 2003–2018.

**Fig 5:**
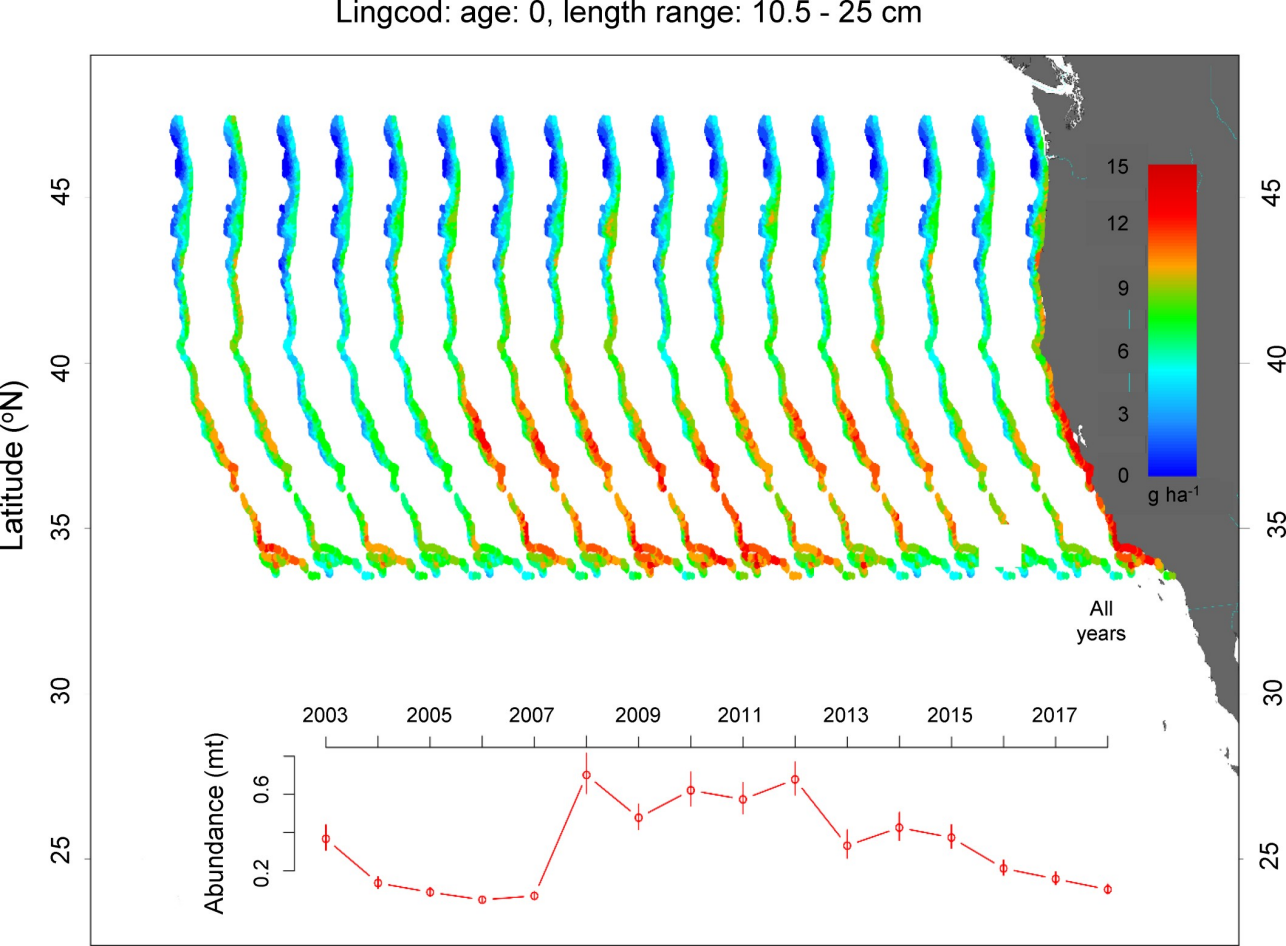
Spatial distribution and annual abundance index of juvenile lingcod. All Years was the average juvenile density from 2003–2018.

For juvenile arrowtooth flounder, sablefish, lingcod, longspine thornyhead, petrale sole, and Pacific sanddab, the areas of high juvenile density were more expansive in area (sablefish, Fig 4; lingcod, Fig 5; others Fig H-K in S1 Appendix). These spatial patterns were generally consistent through time. For example, the density of juvenile sablefish was variable with peaks in density in 2004, 2008, 2010, 2013 and 2016. Spatially, across years, age-0 sablefish (Fig 4) had large, primary hotspots north of Cape Blanco and on either side of San Francisco Bay with a smaller hotspot around Point Conception. These hotspots to the north of Cape Blanco were most obvious in years with high coast-wide density of age-0 fishes (2004, 2008, 2013, and 2016, and to some extent 2017), suggesting that successful coast-wide recruitment relies to a large extent on successful recruitment in northern waters. The exception was 2010 when juvenile density was high in the absence of the northern hotspot, but density was high in the southern region. Age-0 sablefish were found on shelf and upper slope waters (50–475 m).

Additionally, age-0 lingcod had a large, primary hotspot in shelf waters (50–240 m) located from Point Arena to the south of Point Conception (Fig 5). However, there were some small secondary hotspots just north of Cape Mendocino and Cape Blanco. Within years, the prevalence of the large southern hotspot varied and was related to overall density of age-0 lingcod. Again, hotspots were most apparent in years with high juvenile density. For lingcod, the density trend indicated longer-term patterns with low density from 2003–2007, followed by higher juvenile density from 2008–2012, followed again by lower density from 2013 to 2018.

**Fig 6:**
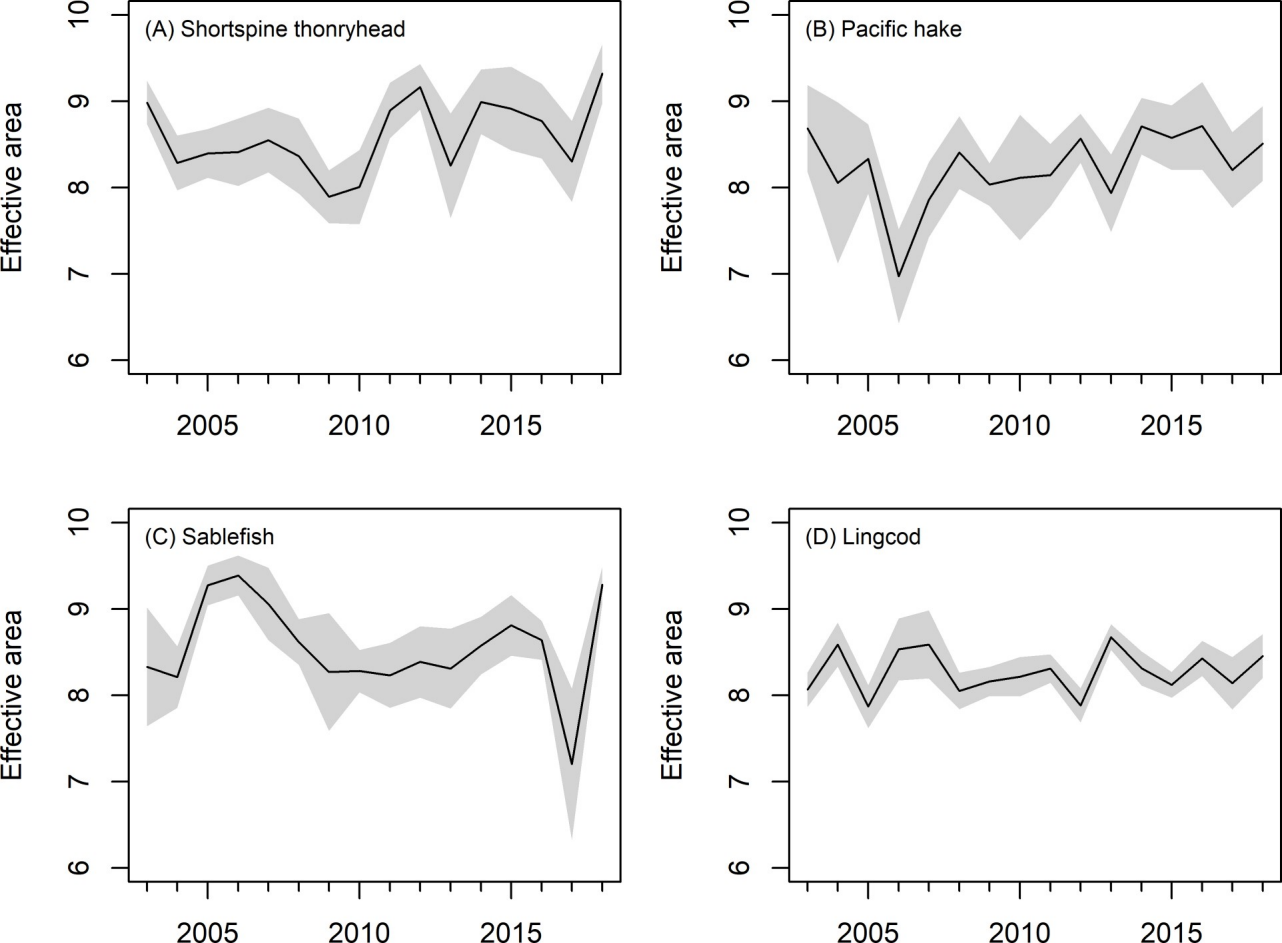
Effective area occupied (1000 km^2^). Grey envelope is +/- 1 s.e.

Effective area occupied varied for all species, but there were no Temporal trends except for Dover sole, which showed a decline in effective area occupied through time (Fig 6 and Fig L in S1 Appendix). Area occupied was negatively correlated with abundance for most species (Fig M in S1 Appendix).

**Fig 7:**
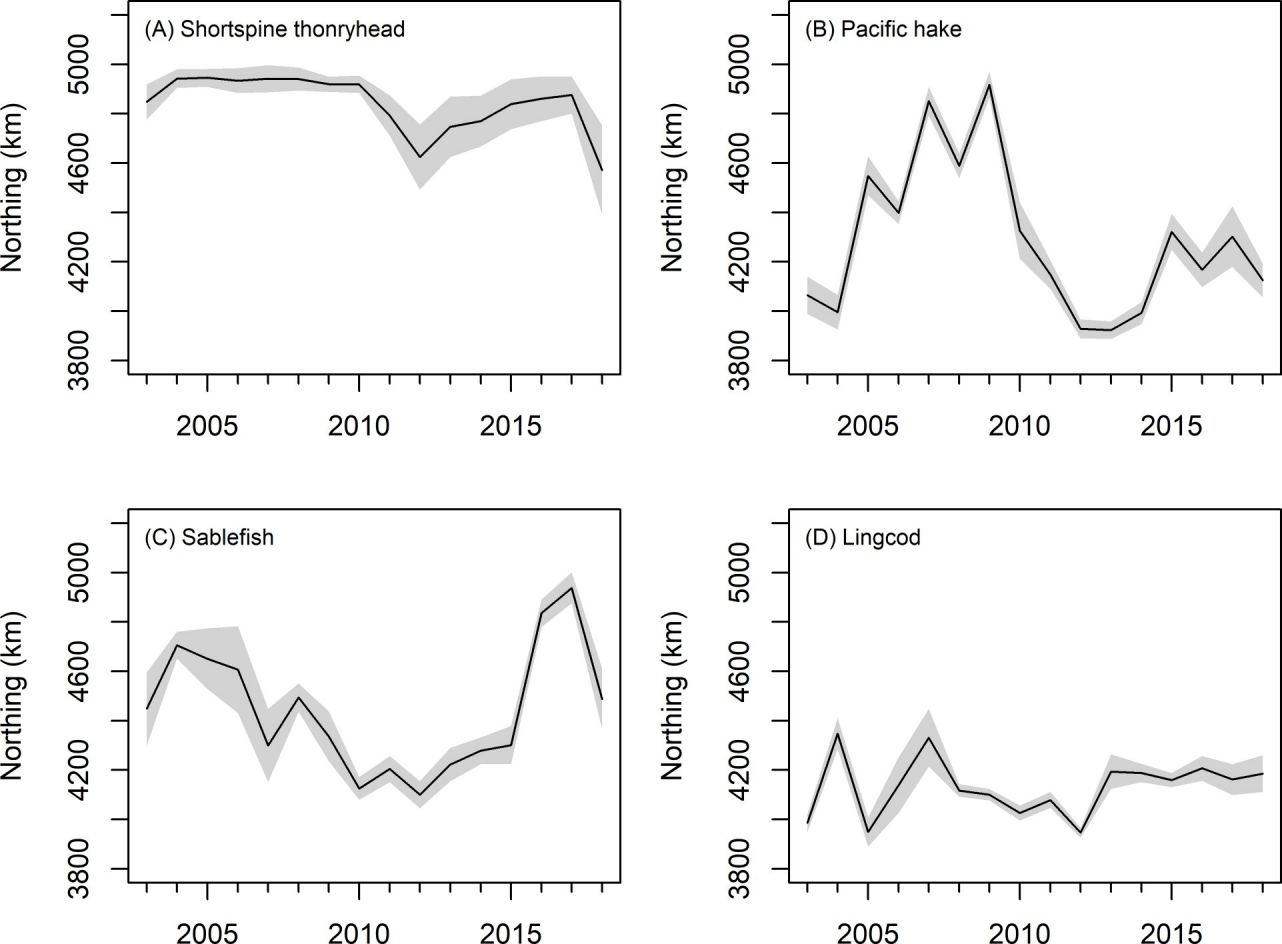
Center of gravity (km from the equator). Grey envelope is +/- 1 s.e.

Two species, Pacific hake and sablefish, showed larger variations in their CoG of approximately 1000 km (Fig 7 and Fig N in S1 Appendix). The remaining species tended to show fluctuations in the range of 200–400 km, but there were no strong directional trends over time except for Dover sole; its CoG shifted south over time (Fig N in S1 Appendix. Relationships between CoG and abundance varied among species (Fig O in S1 Appendix), as did the relationship between effective area occupied and the CoG (Fig P in S1 Appendix). For example, the effective area occupied by Dover sole juveniles decreased as the CoG shifted south over time. However, for longspine thornyhead, higher area occupied was associated with more northerly CoG.

## Discussion

We quantified the temporal variation in the spatial distribution of the juvenile abundance for 13 groundfish species. The observed spatial patterns provide important temporal and spatial information for fisheries managers that will improve our understanding of each species, which was formerly only based on adult distributions. Additionally, we estimated annual coast-wide indices of juvenile density. While evaluating the causes of the observed spatial and temporal patterns was beyond the scope of this paper, we suggest potential ecological and physical habitat mechanisms that likely play a role in the observed temporal and spatial patterns, and we describe how our results have direct application for fisheries management.

Mesoscale geographic features like headlands and submarine canyons can alter local circulation, in particular the development of eddies, which may lead to increased retention of larvae and increased productivity, resulting in higher settlement and better food resources [39–41]. Such processes may explain the location of distinct juvenile hotspots for some particular species and the presence of locations that are hotspots or areas of high juvenile density for many species. For example, multiple species (Dover sole, English sole, hake, lingcod, Pacific sanddab, sablefish and splitnose rockfish) showed high juvenile density near Point Conception, an area of significant eddy formation [39, 41]. Similarly sub-mesoscale eddies form north of San Francisco Bay between Pt. Reyes and Pt Arena [41] where several species also had high juvenile density. Water circulation on wider, shallower sloping areas of the shelf off of the Washington coast is slower leading to higher residence times, and potentially higher retention, which may also help to explain areas of high juvenile density in areas with extensive shelf habitat [39, 42]. Transport mechanisms will obviously interact with an individual species’ life history, in particular the location of any spawning habitats and those advantages to larval biology.

Horizontal transport and water temperature during the pelagic larval stage prior to recruitment to demersal habitat also likely drive the inter-annual variation in juvenile densities seen here [43, 44]. For example, inter-annual recruitment variation around the stock-recruitment relationship for sablefish and petrale sole is associated with horizontal transport and water temperature during specific phases in the pelagic life-history of these species [45, 46]. The current results for sablefish are consistent with stronger age-0 recruitment when conditions favored the onshore transport of eggs and northerly transport of yolk-sac larvae [45]. Recruitment variation in bocaccio rockfishes, *Sebastes paucispinus*, is related to basin scale processes and density dependence [47, 48]. For stocks with weak stock-recruitment relationships and persistent low spawning, inter-annual climate variability is probably the most important driver of recruitment variation [45, 49, 50]. However, for species like lingcod that have experienced recovery in spawning density during the period studied [51], increases in juvenile density over time were also likely due to increased spawning biomass.

Depth is clearly an important factor (as a proxy for other variables such as light, pressure, temperature or oxygen saturation) in determining assemblage structure in marine fishes [52–55], and this pattern was supported by our analyses. Some species, such as English sole, Pacific sanddab, petrale sole, and lingcod had fairly restricted depth distributions of 100–200 m for juveniles on the shelf (Table 1). Others like arrowtooth flounder, Dover sole, sablefish and shortspine thornyheads had broader juvenile depth ranges across the shelf and upper slope. Pacific grenadier and longspine thornyhead had broad depth ranges that encompassed deeper slope waters.

While not examined here, bottom type, biogenic habitat, temperature, frontal boundaries, and other physical characteristics are known to be important in influencing species distributions, community structure, and the location of juvenile habitats [52, 56–58]. For demersal fishes early, post-settlement mortality is often high, density-dependent and mediated via competition for shelter [59]. Bottom depth and bottom temperature are important predictors of recently settled juvenile Kamchatka flounder *Atheresthes evermanni* [58], and the size and location of the cold pool largely determines the distribution of arrowtooth flounder in the eastern Bering Sea [60]. In waters off the U.S. west coast, groundfish assemblages on the slope shift to deeper waters moving north to south likely tracking changes in bottom temperature [52], and geographic features like Point Conception, Cape Mendocino, and Cape Blanco are known transition zones for biogeographic regions in the California Current [52, 53, 61–63].

For semi-pelagic or mid-water species like Pacific hake, characteristics of the water column are habitat, and factors like temperature, currents, and distance from the shelf break may be important in determining both spawning location and the distribution of pelagic juveniles [56]. Immature Pacific hake have higher biomass in cooler than average temperatures, while the response of adults varies with latitude [27]. Additionally, hake tend to be found in areas with poleward drift, which may aid in their annual migration to northern waters [56]. Temporal variability in these factors may explain the variability in the location of hotspots for juvenile hake seen here.

The distribution of adult habitat may also explain some of the observed juvenile hotspots in that juvenile fishes need access to adult habitats as they age. Many groundfishes recruit to shallow habitats, potentially to avoid cannibalism or predation by other species, but move to deeper areas as they grow and age. For example, sablefish show ontogenetic increases in depth distributions [45]. The location of juvenile hotspots for sablefish were inshore of adult areas [64], especially the shelf and upper slope waters between Cape Mendocino and the Columbia River outflow. Successful coast-wide recruitment, in terms of total abundance, appears reliant largely on recruitment to this northern hotspot, which is consistent with onshore transport during the egg stage and northerly long-shore transport during the yolk-sack larval stage in the region being correlated with stronger recruitment [45]. Additionally, the sablefish assessment, which incorporates age structure data across multiple age classes to estimate recruitment history, also captured the high age-0 density years seen in the WCGBTS data [49, 50], suggesting that these northern recruitment pulses were important to stock size. Current habitat maps of EFH for juvenile sablefish [64] show the majority of juvenile EFH in the same area, but in slightly deeper waters farther off shore. Our results suggest that the EFH definition for juvenile sablefish could be revised to include to shallower waters in this northern area.

Several species showed large-scale spatial discrepancies between the distribution of juveniles and adults, raising the question of what ecological processes (or potentially methodological mechanisms) establish adult distributions. For example, shortspine thornyheads had a single, high-density northern juvenile hotspot that was consistent across the study period. However, adults are relatively evenly distributed along the coast at mid- to deeper-depths much further south than this northerly area with a high density of juveniles [29]. Two potential mechanisms could lead to the distribution of adult thornyheads. First, dispersal of young fish from this northern hotspot could lead to a much wider distribution of adult fish farther south. Second, juvenile density was not zero to the south, and consistently lower density in the south could lead to lower density dependence resulting in higher survival in more southerly waters. Other factors such as lower predator densities or differing trawling intensity may also influence the distribution pattern. Likewise, while the density of juvenile Dover sole was highly restricted to several hotspots in the south, adults were widely distributed along the coast. Similarly, splitnose rockfish juveniles occupied several hotspots around and to the south of Point Conception, while adults were abundant around and to the north of Point Conception as well as around and to the north of San Francisco Bay [29], where juvenile density was particularly low.

Contrasting patterns in juvenile and adult biomass were also apparent for lingcod, which are currently assessed as two separate stocks off the US west coast [51]. The most recent stock assessments estimated higher stock biomass off the coasts of Washington and Oregon (21,976 mt) compared to the stock off the coast of California (6,509 mt). However, the distribution of age-0 lingcod in the WCGBT survey was consistently higher south of Cape Mendocino. Longshore movement of age-1+ lingcod does not appear to explain the difference in the distributions of age-0 and adult lingcod as genetic analyses identified genetic differentiation between lingcod north and south of Cape Mendocino [65]. Lingcod initially settle to shallow, vegetated habitats, which the trawl survey (limited to trawlable, non-rocky habitats and depths >55 m) does not sample. So the survey may not adequately describe the distribution age-0 fish if the distribution diverges in these shallower habitats. Alternatively, these genetically dissimilar sub-stocks may have different ecology in the north and south with different levels of recruitment and survival of juvenile fish. Since lingcod move to deeper habitats as they grow and age, the observed distributions are to some extent the result of processes like post-settlement migration, predator avoidance, prey availability and habitat connectivity.

Long-term changes in the latitudinal distribution of fish biomass (CoG) on in the California Current have varied among species [36, 66, 67]. For example, from 1977–2013, the CoG for lingcod, rex sole *Glyptocephalus zachirus*, spotted ratfish *Hydrolagus colliei*, darkblotched rockfish, greenstriped rockfish *S*. *elongatus*, and slender sole *Lyopsetta exilis*, all shifted north—a response that might be expected due to long-term warming—while the distribution of Pacific hake and sablefish biomass moved south [67]. Here, while the juveniles of some species like Pacific hake and sablefish showed large fluctuations in their CoG form 2004–2018, only Dover sole had a significant shift in biomass of juvenile fishes to the south. Instead, the variability in the latitudinal distribution appeared related to the fluctuation in the intensity of juvenile hotspots. For example, when sablefish had high age-0 abundance in the north, the CoG shifted north [68].

The species distribution maps presented here should be interpreted in relation to methodological factors like gear type, survey design, and catchability [69, 70]. The trawl survey samples between approximately 55–1280 m, and any conclusions are limited to trawlable (non-rocky) habitat in this range. For some species, like lingcod discussed above, this limited depth range may explain mismatches between juvenile abundance patters and adult biomass. Additionally, because the trawl survey does not sample complex, rocky habitats, abundance estimates may be biased for some species, especially if there is density dependence [60, 71–73].

For many species, the effective area occupied was negatively correlated with abundance, albeit the relationship was not significant in all cases. The relationship does not mean that the latitudinal or depth range of the species contracted at higher juvenile biomass. Effective area as calculated by the VAST package is the area needed to contain the population at average biomass-density [30]. Therefore, this relationship would be expected as densities increase in hotspots more quickly relative to other areas, thus concentrating biomass–a pattern we might expect if juveniles follow ideal free distributions [74–76]. Rockfish, for example, are generally more abundant on complex, rocky habitat. Our species distribution maps likely, therefore, miss or underestimate biomass in juvenile hotspots associated with untrawlable habitat like rocky reefs or rocky pinnacles. Temporally, if juvenile abundance follows an ideal free, trawl-derived abundance indices may underestimate abundance at low densities because densities will increase in the primary and unquantified habitat (untrawlabe) before increasing in secondary, sampled habitat (trawlable). Conversely, at higher levels, juvenile density will continue to increase in trawlable areas after density in untrablable areas has stabilized, potentially overestimating abundance. Nevertheless, for species like sablefish, the observed temporal patterns largely match model derived trends for age-0 abundance in the stock assessments.

Gear selectivity in relation to fish body size may also influence the results to some extent. For many species, we had to combine age classes to obtain enough data to produce acceptable model fits. Growth of recently settled individuals over the sampling period may influence the distributions as larger fish become more selected. However, the survey is conducted in two passes between mid-May and October, and we account for this effect in part by including pass in the VAST model. Including pass also accounts to some extent for variability in the within-year timing of settlement. These effects are most likely more important for species where we analyzed a single age class. For each pass, vessels begin in Newport, Oregon and initially work north to Cape Flattery; vessels then head south to San Diego [34]. The timing of sampling within year could, therefore, miss late-season settlement in northern waters if settlement occurs after the vessels have passed. Natural mortality may lead to lower estimates of abundance within years for southern waters, as fishes in the south well have been subjected to more predation or starvation risk when they are caught comparted to more northerly fish.

Catchability, the product of the efficiency of the sampling gear and the availability of taxa to that gear [69, 70, 77], likely varies among depths, bottom types, and the size of individuals among other factors. Since we have not adjusted for catchability, the estimates of abundance here should be considered indices and not absolute measures of biomass. While the effect of different sediment sizes within the range of trawlable habitats may not be large [70], the spatial patterns may be influenced by these factors and the distribution maps should be interpreted with this issue effect in mind.

Species distribution modes have been increasingly used to define essential fish habitat in regions managed by the U.S. National Marine Fisheries Service [58, 78], as mandated by the Magnuson-Stevens Act. For example, Laman et al. [58] estimated habitat-based models for Alaskan species. Our analyses increase the information available to fisheries management practices of the 13 stocks we analyzed. The spatial and temporal results of this study can be used as leading indicators of potential future changes in stock size for both stock and integrated ecological assessments [45, 79] or for investigating environmental drivers of recruitment variation, particularly for those results that capture age-0 and age-1 fish. Delineation of juvenile habitats can also help to parameterize dispersal models and understand dispersal patterns and connectivity [78]. If fact, one of the motivators for the present study was the need to identify juvenile habitats for petrale sole for use in an individual-based dispersal model.

Fishery managers can use the spatially explicit information on juveniles from our analyses to further refine and improve essential fish habitat and nursery habitat definitions and delineations. Identifying juvenile habitats allows for more targeted spatial management and conservation, allows for better prediction of responses to environmental events, and forms the foundation for a more complete understanding of a species ecology [78]. For example, fisheries closures implemented to protect adult rockfish and adult habitats led to increased spawning output near closed areas [10]. In the North Sea, managers implemented a closed area to protect the nursery habitat of North Sea plaice *Pleuronectes platessa* from trawling [26]. However, the closure of plaice nursery habitat to trawling was unsuccessful because the distribution of juvenile plaice shifted. The locations of juvenile hotspots for the species we analyzed were generally persistent through time, suggesting that closures or gear restrictions to protect juveniles and juvenile habitat might be effective mechanisms to protect juveniles for these species. Since many species show ontogenetic shifts to deeper waters, such spatially explicit and targeted closures might not adversely affect adult fishing effort, although adverse impacts will be dependent on gear type and the species’ life history, since one species’ adult habitat may be another’s juvenile habitat. Likewise, the multi-species nature of the fishery may also complicate spatial management, but good information on the HAPC for target species is essential for resolving such issues.

Our characterization of the spatial distribution of juvenile habitats improves our understanding of how climate or physical conditions affect juvenile survival. For example, there is a large oxygen minimum zone (OMZ) in mid-depth (500–720 m) slope waters off the NW Pacific Coast of the U.S. [80]. This OMZ affects both assemblage structure [52] and diversity [53], and there is evidence that dissolved oxygen (DO) concentrations are decreasing in the OMZ [81, 82]. Shoaling of these low DO waters can lead to fish kills and alter the behavior and occurrence of fishes [80, 82]. Knowledge of the location of juvenile habitats will aid in assessing the vulnerability of these species to low DO events.

## Conclusions

Our analysis represents a refinement of information available for the delineation of essential fish habitat for west coast groundfishes by identifying, more specifically, the geographic locations and temporal stability of juvenile habitats (and potential nursery areas). As we address only the density of juvenile fishes, the areas we identify are best termed juvenile habitat rather than nursery habitat. Nevertheless, these areas represent potential, if not likely, nursery habitat for some species. Further work such as tagging studies or growth analyses will help us to better understand whether these areas function as nurseries and their importance to the population dynamics of each species.

## Supporting information

S1 Appendix (PDF)

S2 Appendix (PDF)
