# Supplementary material for: Spatio-temporal patterns in juvenile habitat for 13 groundfishes in the California Current Ecosystem
Source: PLoS One. 2020 Aug 21;15(8):e0237996. doi: 10.1371/journal.pone.0237996 (PMC7442253; doi:10.1371/journal.pone.0237996)
Supplement: S1 Appendix — (PDF) [file pone.0237996.s001.pdf]

S1 Appendix Supporting Information

Data Selection

Table A. Sablefish length distribution by age class.

	Length (cm)																
**Age**	**17**	**18**	**19**	**20**	**21**	**22**	**23**	**24**	**25**	**26**	**27**	**28**	**29**	**30**	**31**	**32**	**33**
Age-0	5	1	8	18	40	57	77	103	117	93	56	26	**10**	**6**	1	0	0
Age-1	0	0	0	0	0	0	0	0	0	1	2	1	**9**	**11**	20	56	85
Age-2	0	0	0	0	0	0	0	0	0	0	0	0	0	0	0	0	0

The table shows a subset of sablefish length-at-age data. We set maximum length for an age class as one length bin below the point where were more older than younger aged individuals in that length bin. For sablefish, this transition occurred between 29 and 30 cm; the 30 cm length bin is the first to contain more age-1 (11) than age-0 (6) fishes. Therefore, we set the maximum size of age-0 fishes as 29 cm.

VAST

Table B shows the parameters settings used in the VAST analyses. For more detail on VAST see Thorson [1].

Due to the large number of species we examined, and because the behavior of fit statistics like Akiake's Information Criterion are not well understood for this class of models, we did not conduct extensive sensitivity analyses on the modeling results. Instead, we chose model parameters and specification based on biological assumptions (e.g., the choice of a single intercept across years) or previous modeling work (e.g., the choice of the Poisson-link function). We did examine model-fit diagnostics for each species (e.g., examine residuals, positive definite Hessian). Additionally, we did not examine the effects of increasing the number of age classes included in the analyses beyond including sufficient data for obtaining good model fits. Here, we were interested in quantifying the

distributions of juvenile fishes with the goal of identifying recruitment habitats. Therefore, we included only the youngest age classes or smallest sizes required to achieve stable model fits. Model diagnostics (QQ plots and spatial residual plots) can be found in S2 Appendix.

Table B. VAST model parameters.

VAST Parameters	Juvenile Model
Data	WCGBTS
Years	2003 – 2018
Dependent Variable	Biomass per swept-area
Prediction Variables	Year of Capture, Vessel, & Tow Location (Latitude and Longitude)
Density covariates (used to improve interpolated/extrapolated predictions of density)	Depth
Method	Mesh
Region	Other (extrapolation grid defined by the data extent for each species).
Knots	600
Longitudinal Limits	Varies by species
Depth Range	Varies by species
Field Config	Omega1 = 1, Epsilon1 = 1, Omega2 = 1, Epsilon2= 1
Rho Config	Beta1 = 0, Beta2 = 0, Epsilon1 = 0, Epsilon2 = 0
Overdispersion Config	Delta1 = 1, Delta2 = 1
Obs Model	ObsModel = c(2,1)<br><br>Gamma-distribution errors for the positive catch rates with a "Poisson-link" function that approximates a Tweedie distribution

See Table 1 for depth ranges. See Thorson [1] for an extensive discussion of choosing parameter settings for VAST.

Results

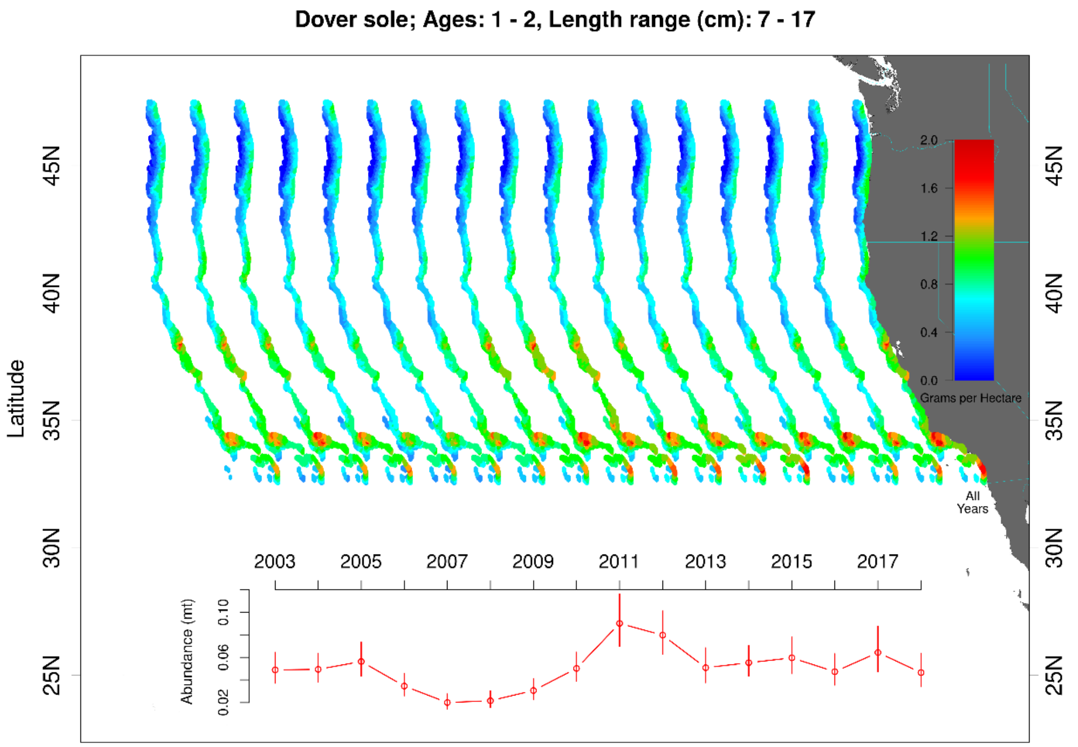

Fig A. Spatial distribution and annual abundance index of juvenile Dover sole. All Years was the average juvenile density from 2003-2018.

Dover sole showed across-years juvenile (age-1 and age-2) hotspots in four locations: just north of San Francisco Bay, in Monterey Bay, just south of Point Conception, and off of San Diego (Fig A). Annually, the hotspot on the southern side of Point Conception was the most consistently present, while hotspots at other locations varied temporally with the overall abundance of juveniles. For example, the hotspot north of San Francisco bay was only evident in the 2011 and 2012 when juvenile abundance was high. However, this location did not have a hotspot in 2017 when juvenile abundance as also high. Dover sole juveniles were more commonly found in shallower shelf and upper slope waters (50-465 m).

English sole; Age: 1, Length range (cm): 9 - 16

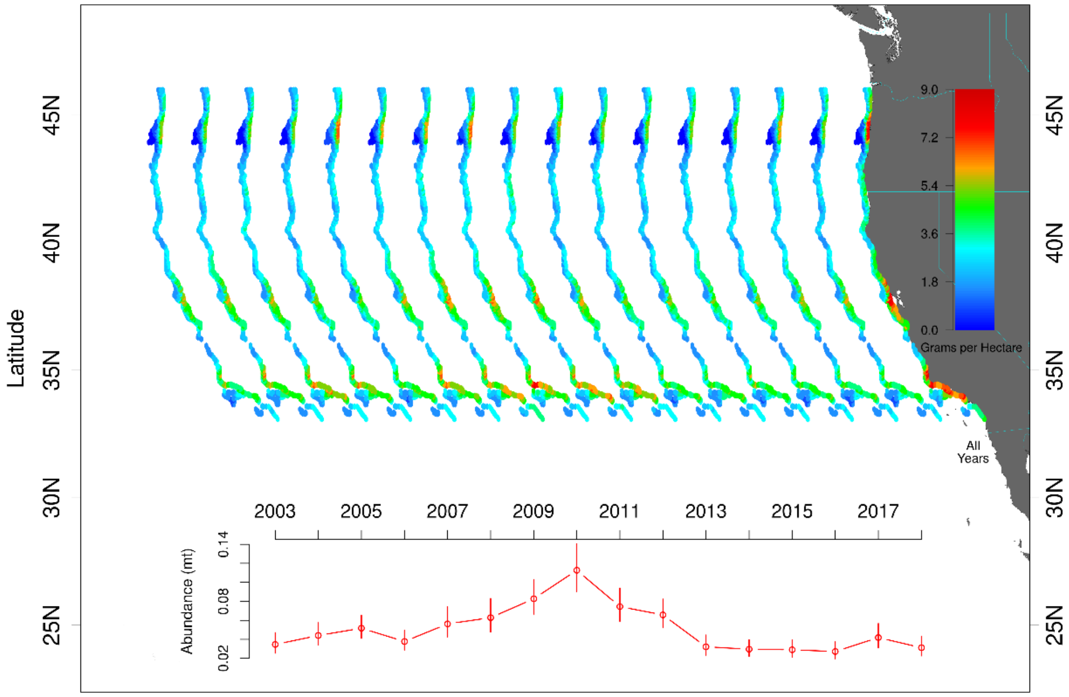

Fig B. Spatial distribution and annual abundance index of juvenile English sole. *All Years was the* average juvenile density from 2003-2018.

English sole juveniles (age-1) had primary, across-years hotspots in shelf waters (50-140 m) at three locations: at approximately 44 ◦ N on the shelf near Heceta Bank, around San Francisco Bay and on the southern side of Point Conception (Fig B). Annually, hotspots were evident primarily in the late 2000's when age-1 fish were most abundant.

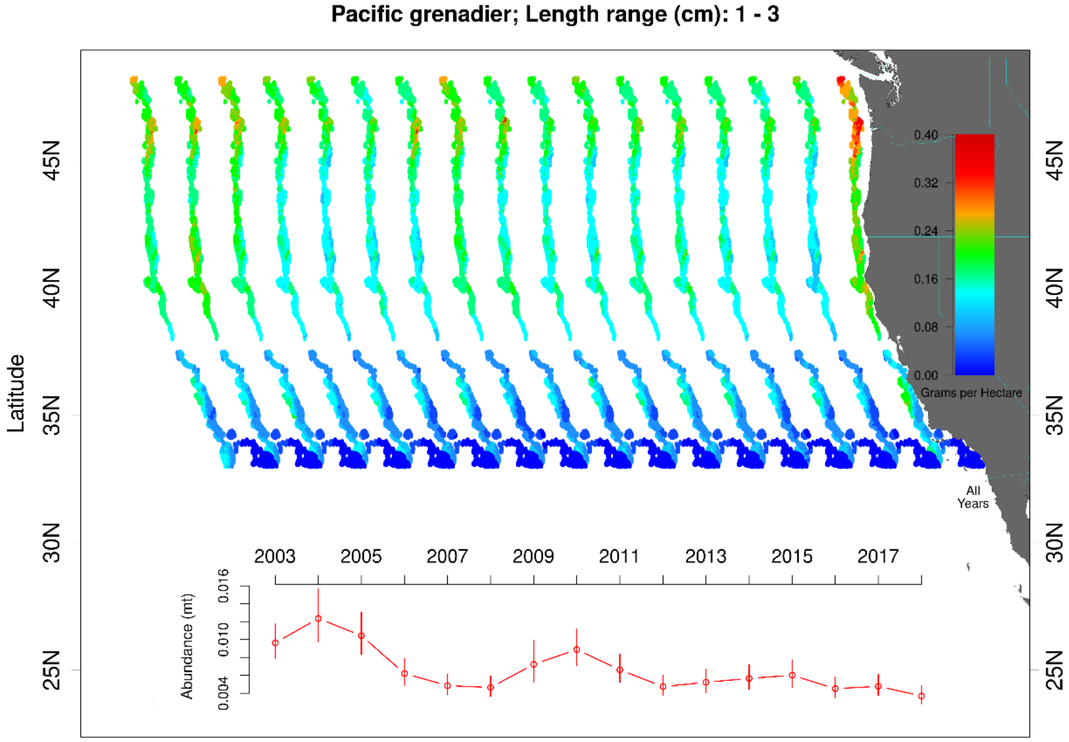

Fig C. Spatial distribution and annual abundance index of juvenile Pacific grenadier. All Years was the average juvenile density from 2003-2018.

Pacific grenadier were most common in lower slope waters (490 – 1275 m, Table 1) north of San Francisco Bay, especially off the Columbia River outflow north to Cape Flattery (Fig C). Within years, hotspots occurred in this area in years with high grenadier abundance, when grenadier also spread farther south albeit in lower density (e.g., 2004 & 2005). Across years deeper areas north of Cape Mendocino had the highest juvenile densities.

Splitnose rockfish; Ages: 0 - 1, Length range (cm): 4 - 10

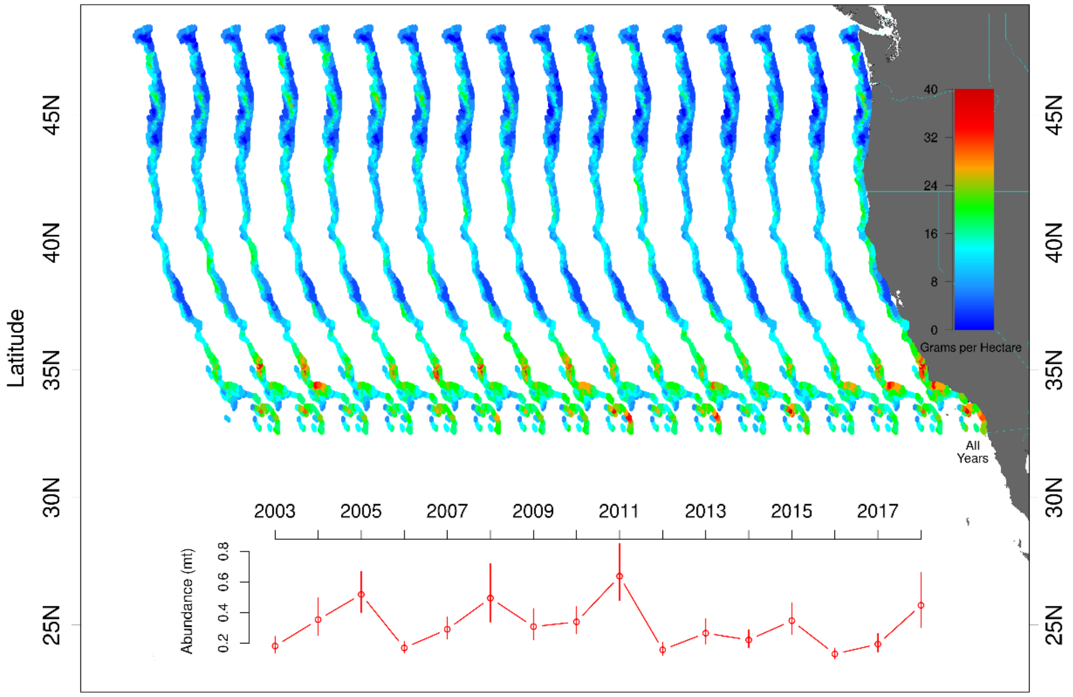

Fig D. Spatial distribution and annual abundance index of juvenile splitnose rockfish. All Years was the average juvenile density from 2003-2018.

Splitnose rockfish juveniles (age-0 and age-1) were most common south of Monterey Bay at 160-625 m. There were across-years hotspots around Point Conception, on the east side of Santa Catalina Island and on the coast just north of San Diego (Figs D and E). These hotspots were comparatively small and most apparent in years when abundance was high.

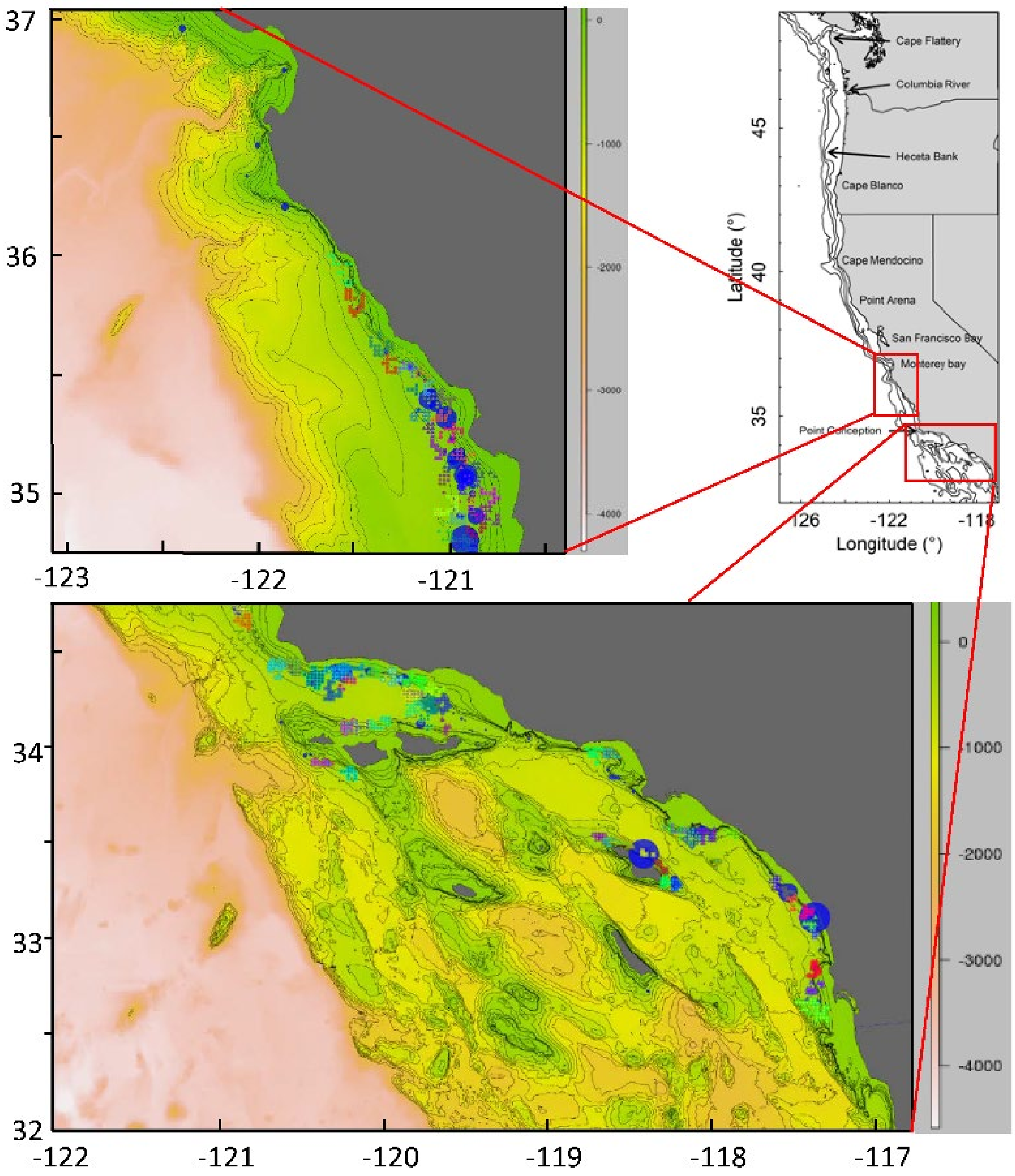

Fig E. Distribution of catch of splitnose rockfish south of Monterey Bay and in the Southern California Bight.

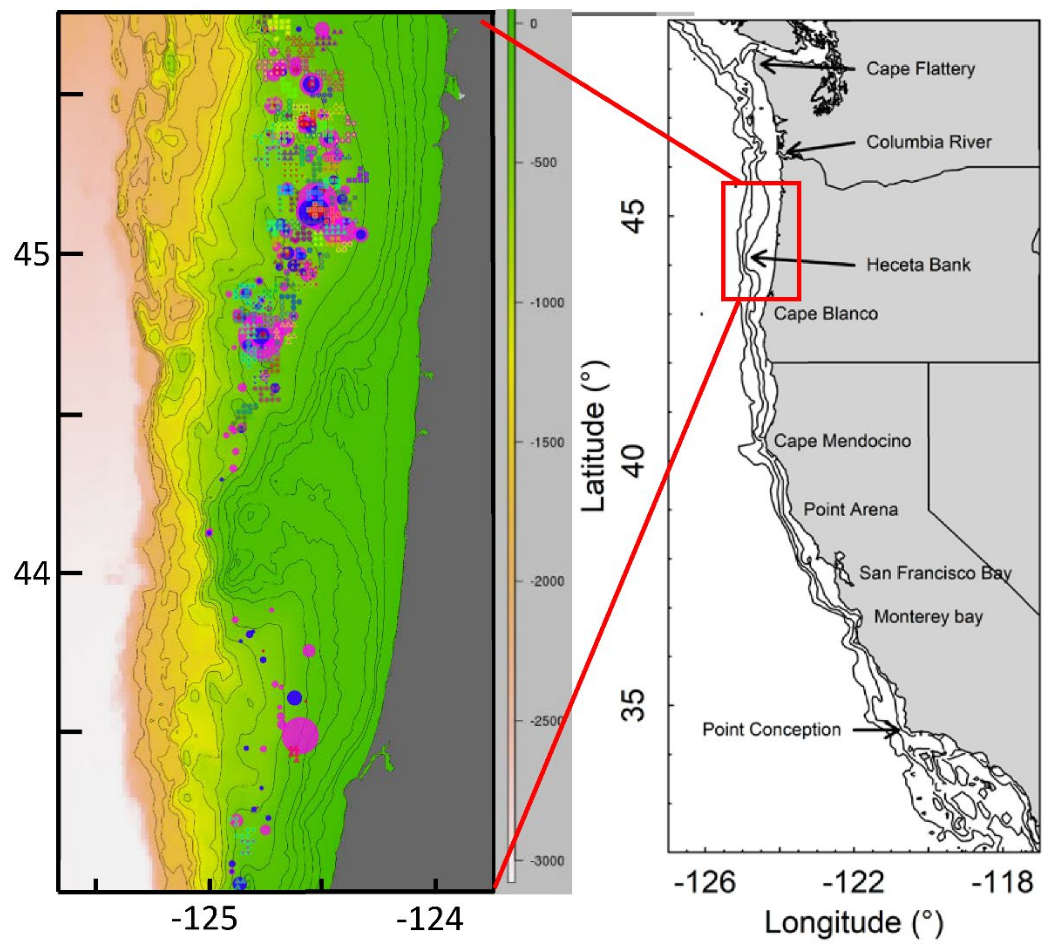

Fig F. Catch of shortspine thornyheads in the vicinity of Heceta Bank.

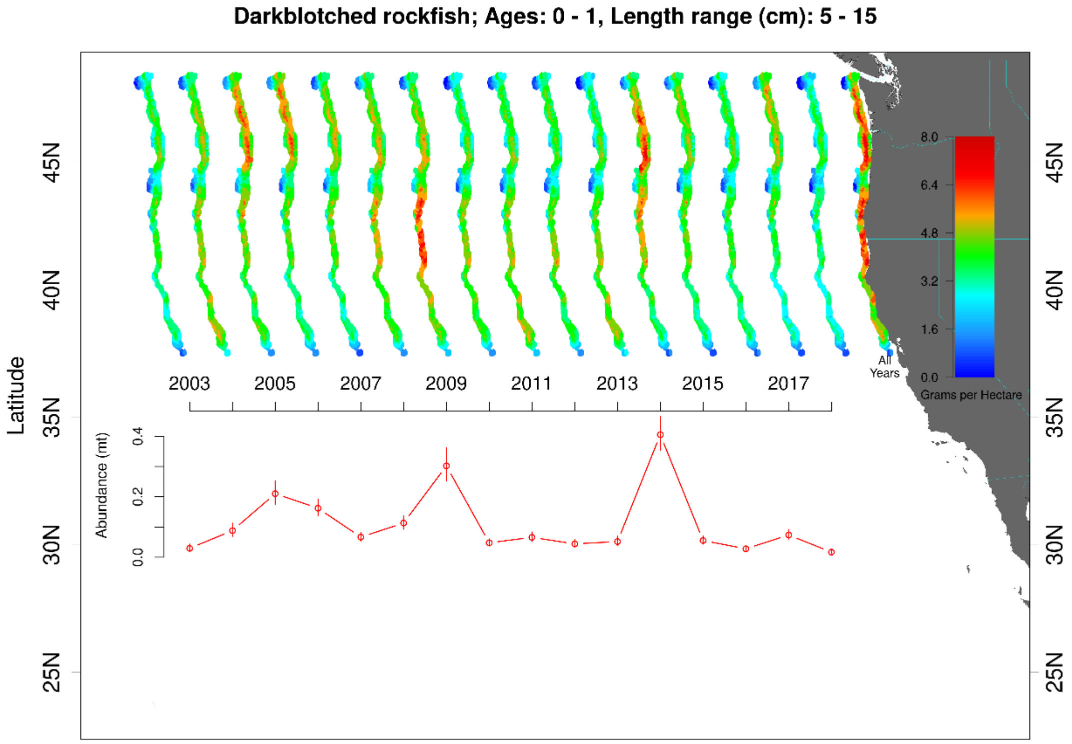

Fig G. Spatial distribution and annual abundance index of juvenile darkblotched rockfish. All Years was the average juvenile density from 2003-2018.

Darkblotched rockfish (age-0 and age-1) were found primarily on the shelf at depths of 80-240 m. There were juvenile hotspots in two general areas that varied from year to year: north of the Heceta Bank area to Cape Flattery and south of Heceta Bank to Cape Mendocino (Fig G). Hotspots were evident when juvenile abundance was high, but the location of the hotspots was not important in determining overall density. For example, hotspots occurred in different areas in 2009 and 2014 when the abundance index was high.

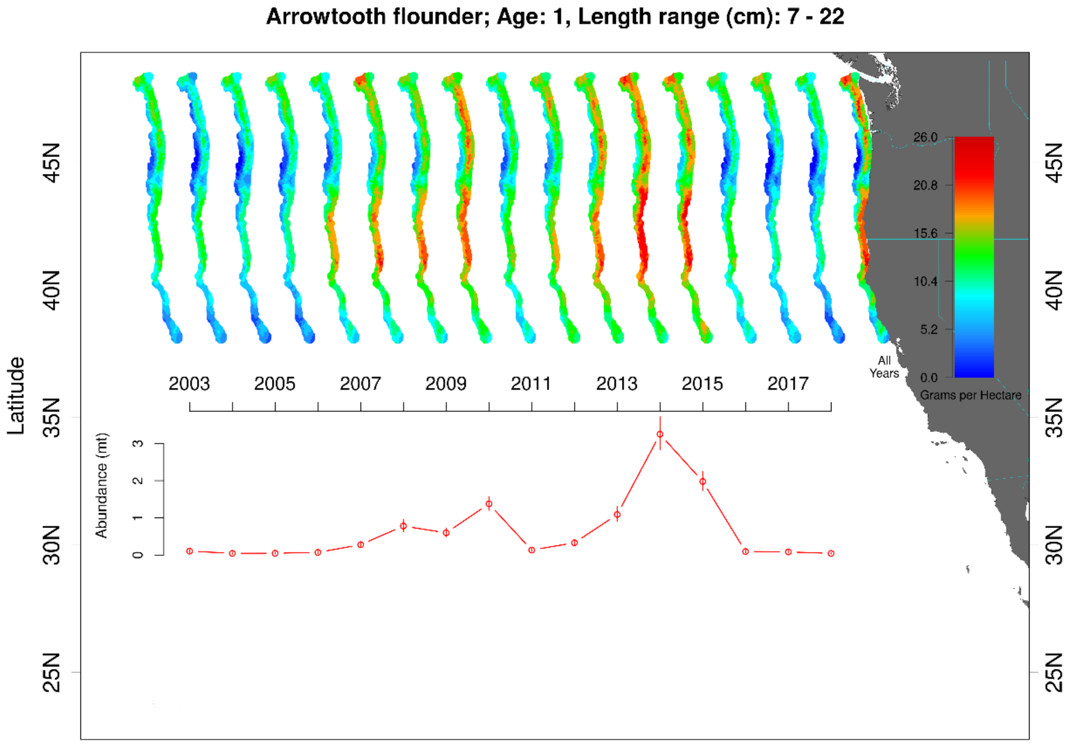

Fig H. Spatial distribution and annual abundance index of juvenile arrowtooth flounder. All Years was the average juvenile density from 2003-2018.

Across years, age-1 arrowtooth had the highest density at Cape Mendocino and Cape Blanco at depths of 50-470 m (Fig H). There was also less consistent but large hotspot to the north around the Columbia River outflow and off Cape Flattery in years when arrowtooth abundance was highest (2010, 2014 & 2015).

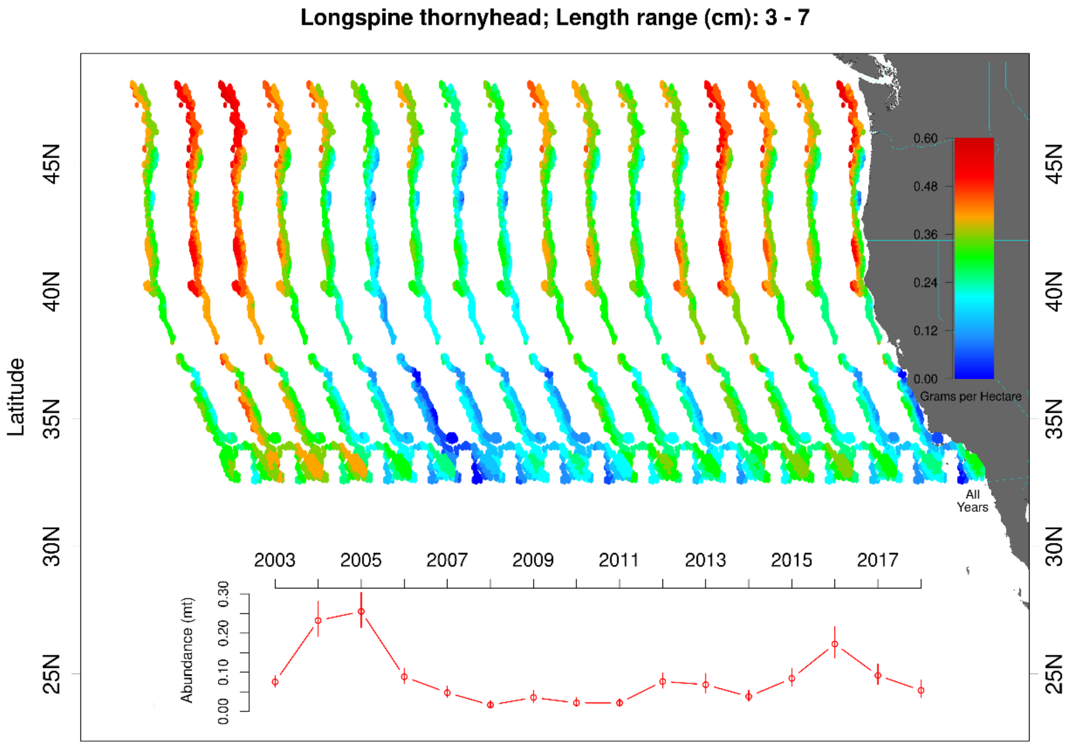

Fig I. Spatial distribution and annual abundance index of juvenile longspine thornyhead. All Years was the average juvenile density from 2003-2018.

Longspine thornyhead juveniles were most abundant on the slope (385-1245 m) from Cape Flattery south to Cape Mendocino across all years (Fig I). Annually, this hotspot was evident in years with high abundance but not in other years. In years with high abundance (2004 and 2005), they were also primary and secondary hotspots between Monterey Bay and Point Conception and around San Diego.

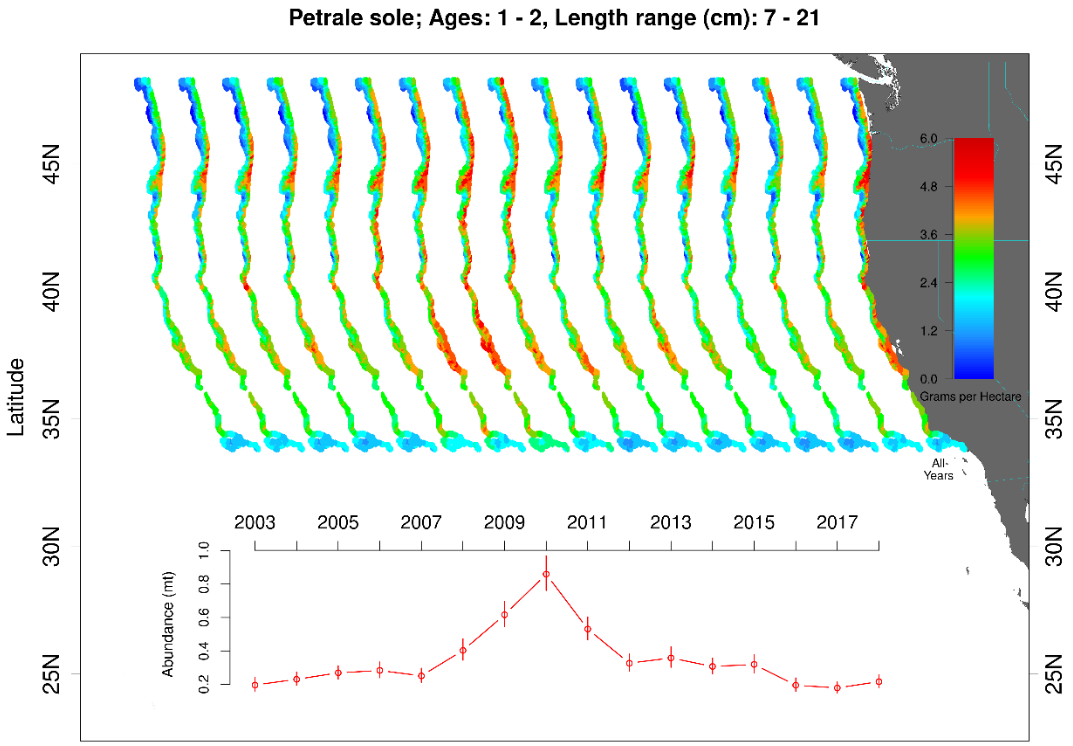

Fig J. Spatial distribution and annual abundance index of juvenile petrale sole. All Years was the average juvenile density from 2003-2018.

Across years, petrale sole (age-1 and age-2) were widely distributed with multiple smaller hotspots on the shelf (50=200 m) north of Monterey Bay (Fig J). The shelf near Heceta Bank and around the mouth of San Francisco Bay had the largest primary hotspots. There were typically hotspots at these locations as well, but in years with high abundance of petrale juveniles (2009-2011) areas with high juvenile abundance expanded to the shelf between Cape Blanco and San Francisco Bay.

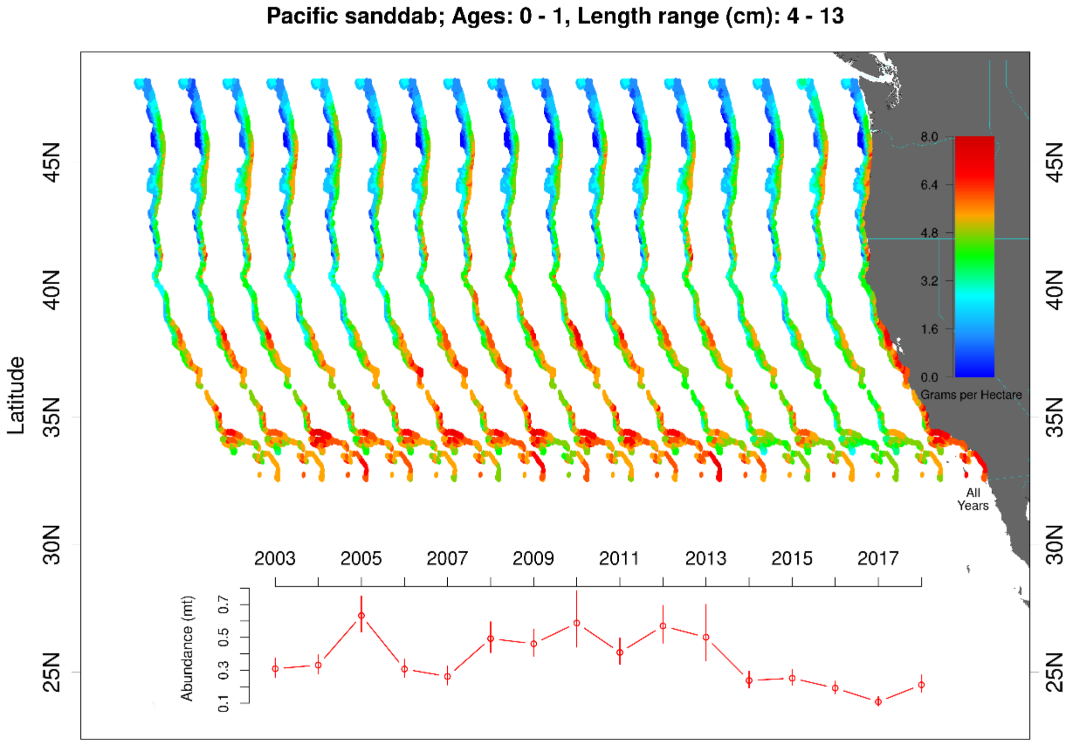

Fig K. Spatial distribution and annual abundance index of juvenile Pacific sanddab. All Years was the average juvenile density from 2003-2018.

Pacific sanddab juveniles were most abundant south of Point Arena in most years with large hotspots around San Francisco Bay and from Point Conception to San Diego (Fig K). They were present at intermediate density on the shelf just north of Cape Mendocino and Cape Blanco in years with high overall juvenile abundance, which resulted in a secondary across-years hotspot in these areas.

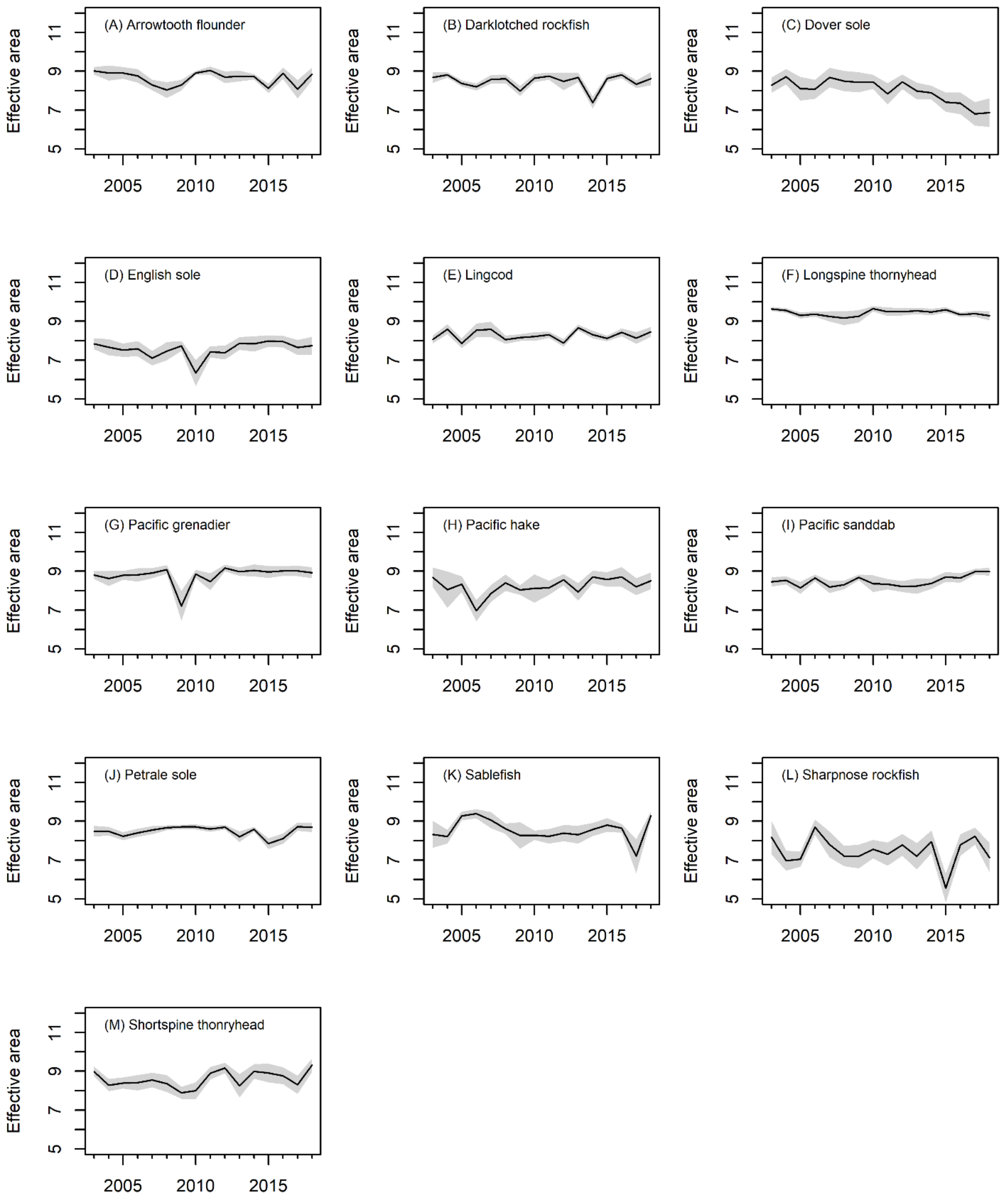

Fig L. Effective area occupied (x1000 km²). Effective area occupied is the area needed to contain the population at average biomass-density, estimated from the VAST model [1, 2].

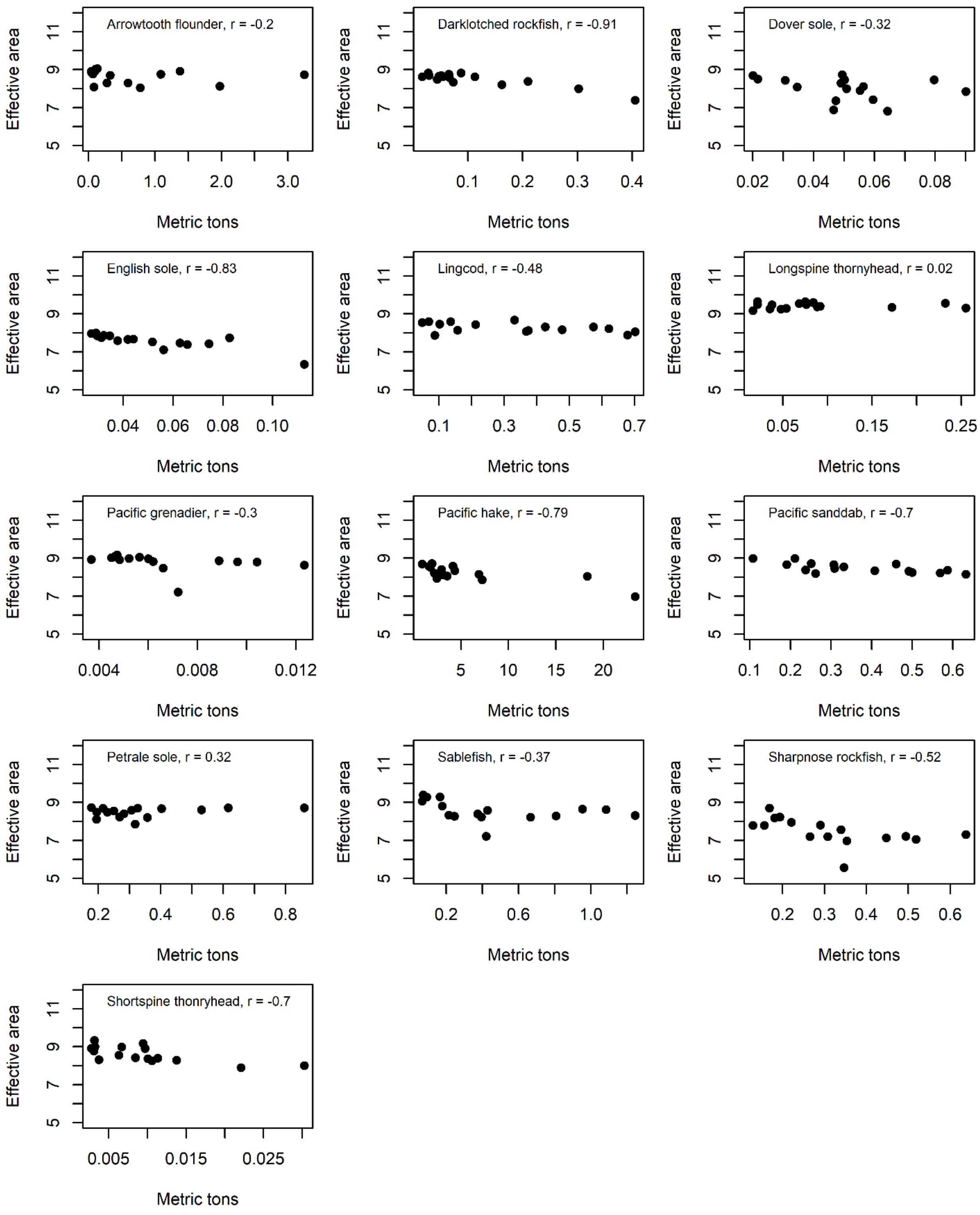

Fig M. Effective area occupied (x1000 km²) and juvenile abundance. Effective area occupied is the area needed to contain the population at average biomass-density. Both are estimated from the VAST model.

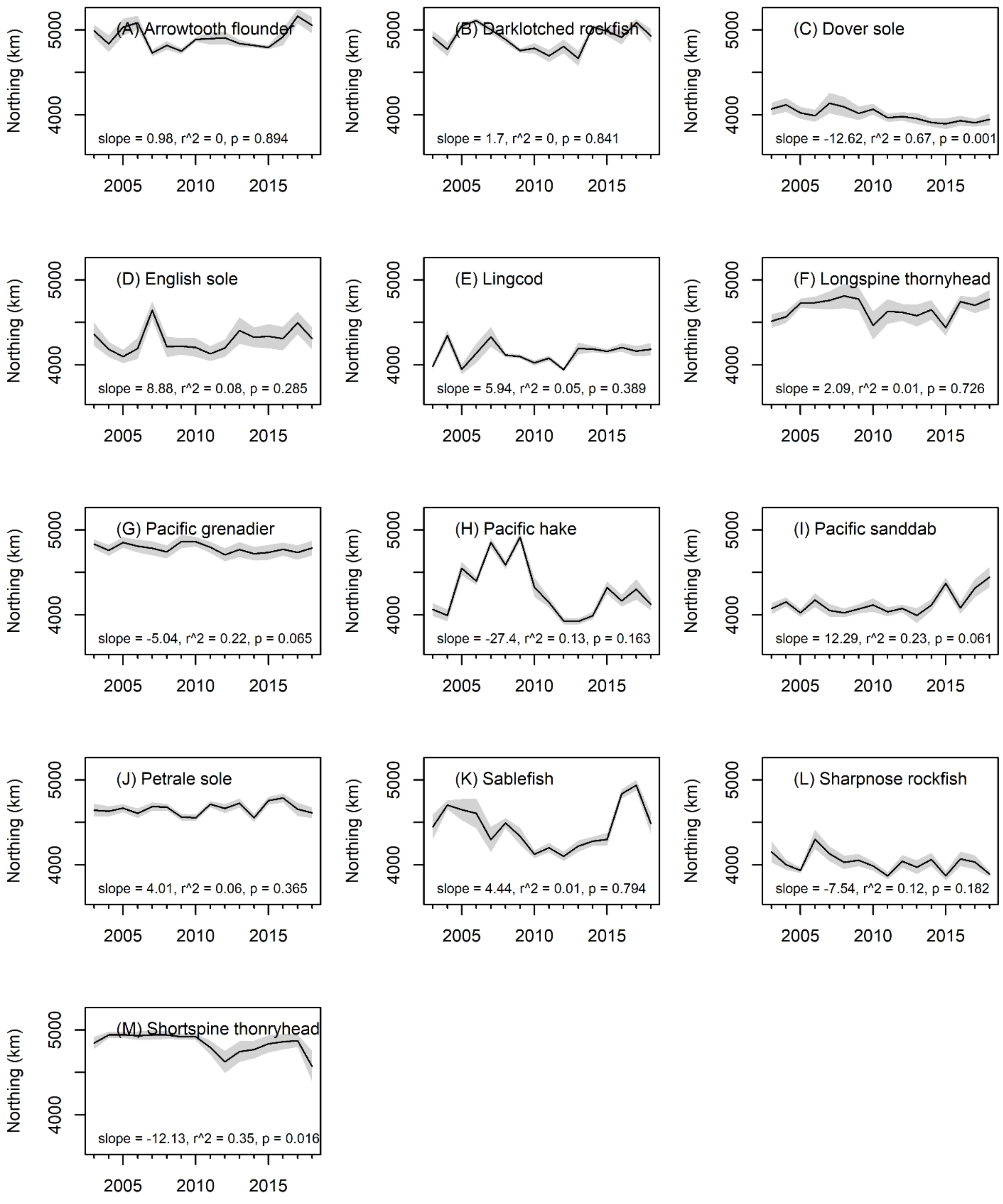

Fig N. Northing center of gravity (km north of the equator). The center of gravity is the latitudinal centroid, estimated from the VAST model. Statistical output are from simple linear regressions between northing and year, weighted by 1/s.e. of the northing estimate.

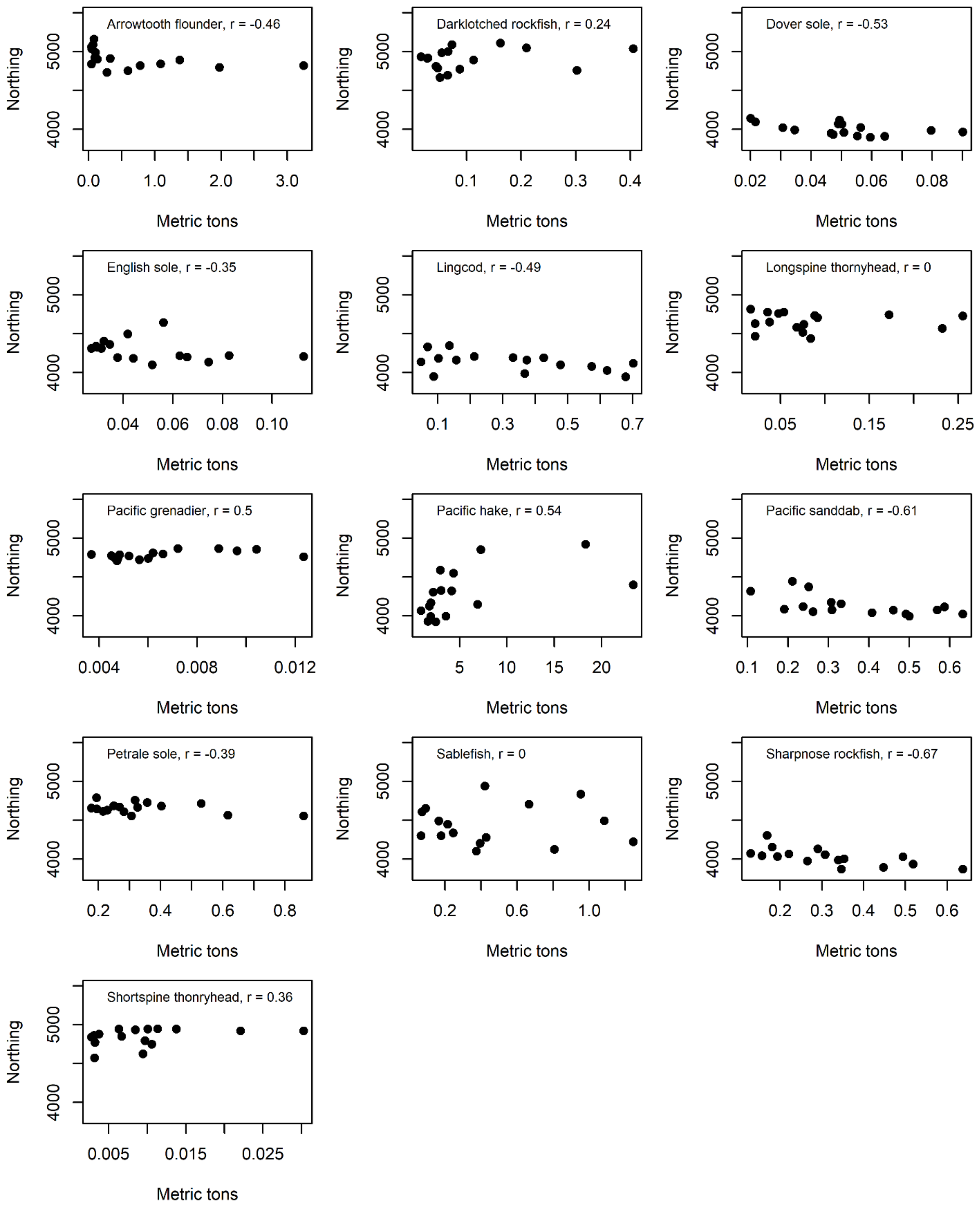

Fig O. Northing center of gravity (km north of the equator) and juvenile abundance. The center of gravity is the latitudinal centroid. Both are estimated from the VAST model.

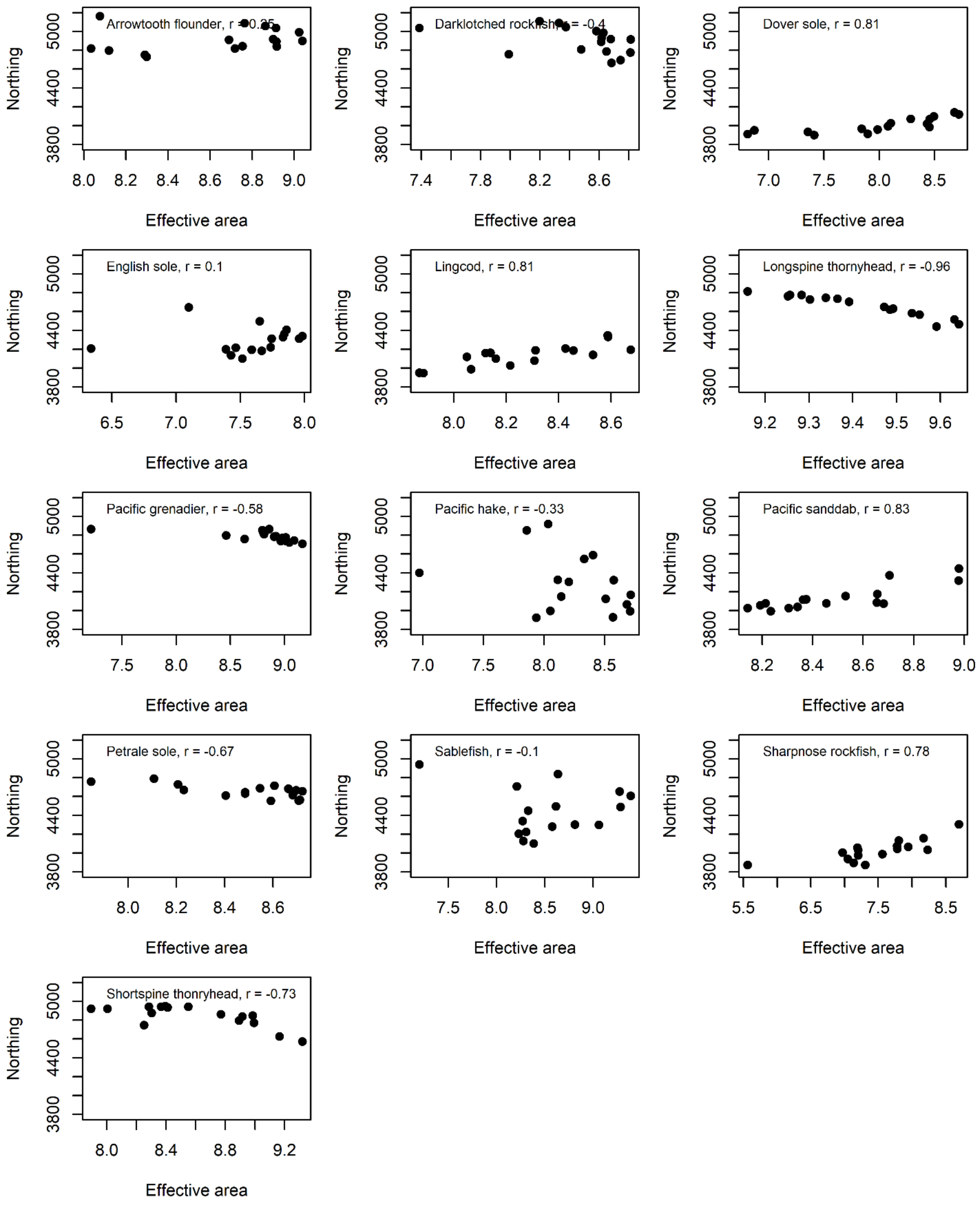

Fig P. Effective area occupied (x1000 km^2) and northing center of gravity (km from the equator). Both are estimated from the VAST model.

Literature cited

1. Thorson JT. Guidance for decisions using the Vector Autoregressive Spatio-Temporal (VAST) package in stock, ecosystem, habitat and climate assessments. Fisheries Research. 2019;210:143-61.
2. Thorson JT, Barnett LAK. Comparing estimates of abundance trends and distribution shifts using single- and multispecies models of fishes and biogenic habitat. ICES J Mar Sci. 2017;74(5):1311-21.
