# Supplementary material for: Spatio-temporal patterns in juvenile habitat for 13 groundfishes in the California Current Ecosystem
Source: PLoS One. 2020 Aug 21;15(8):e0237996. doi: 10.1371/journal.pone.0237996 (PMC7442253; doi:10.1371/journal.pone.0237996)
Supplement: S2 Appendix — (PDF) [file pone.0237996.s002.pdf]

S2 Appendix Diagnostic plots from fitted VAST models

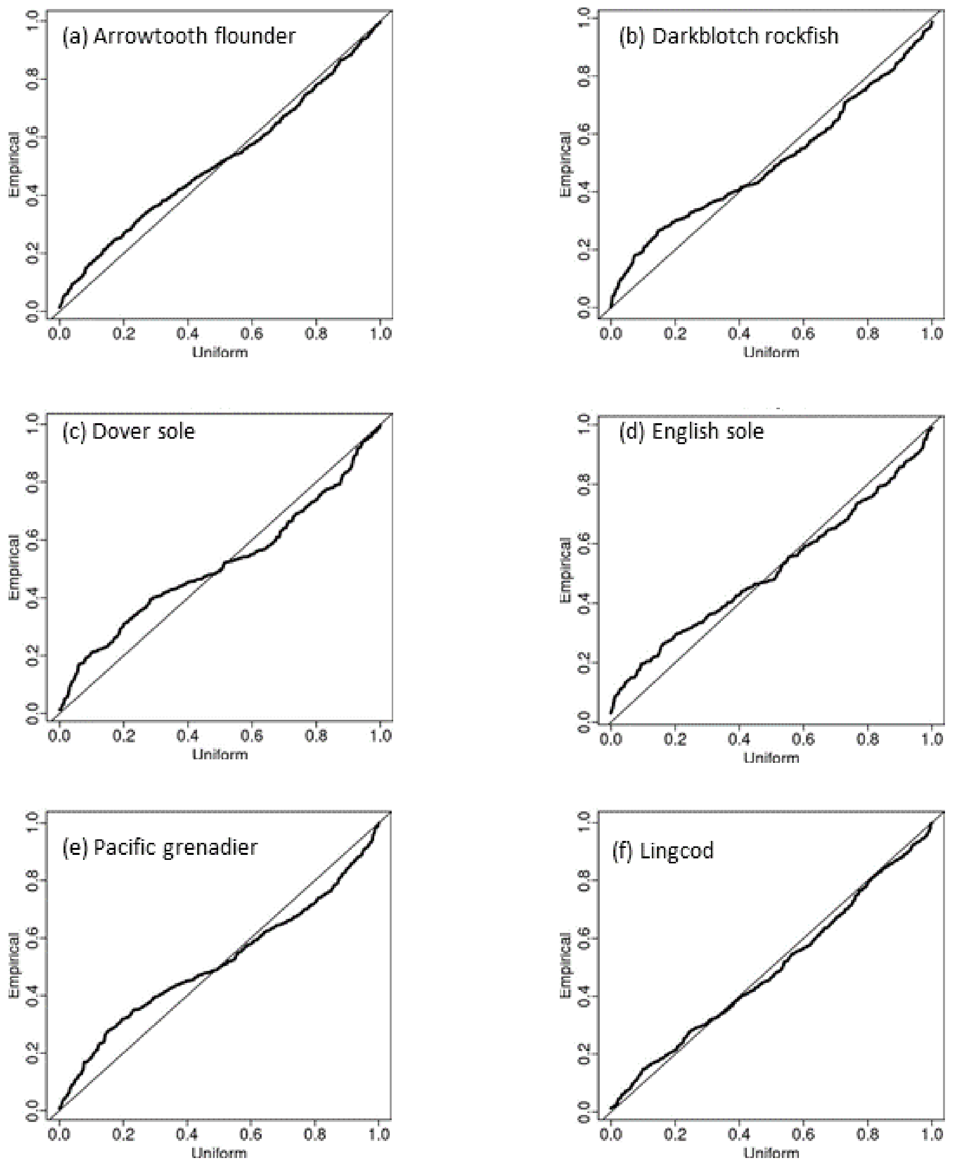

Fig AA. QQ residual plots for arrowtooth flounder, darkblotched rockfish, Dover sole, English sole, Pacific grenadier, and lingcod.

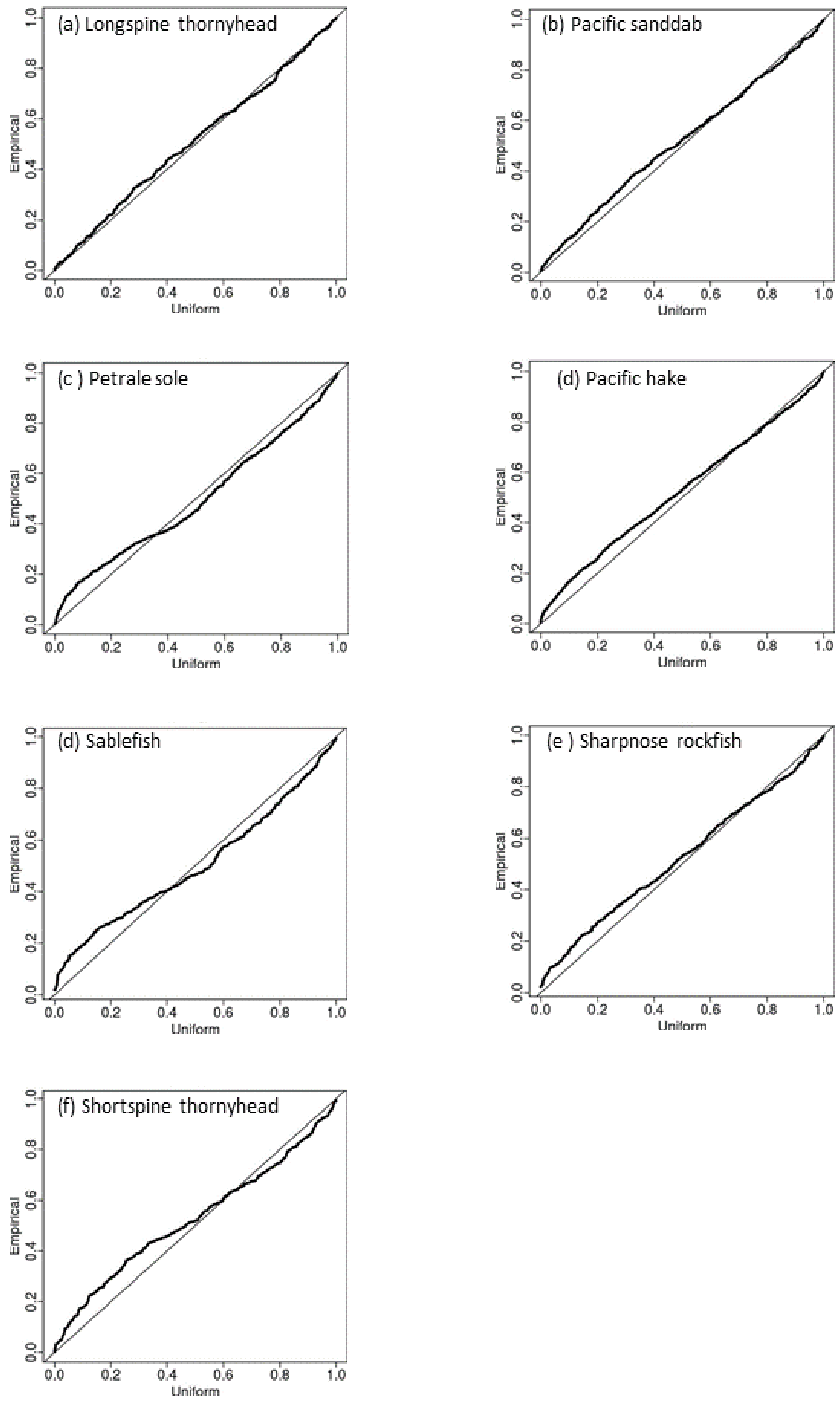

Fig BB. QQ residual plots for longspine thornyhead, Pacific sanddab, petrale sole, Pacific hake, sablefish, sharpnose rockfish, and shortspine thonryhead.

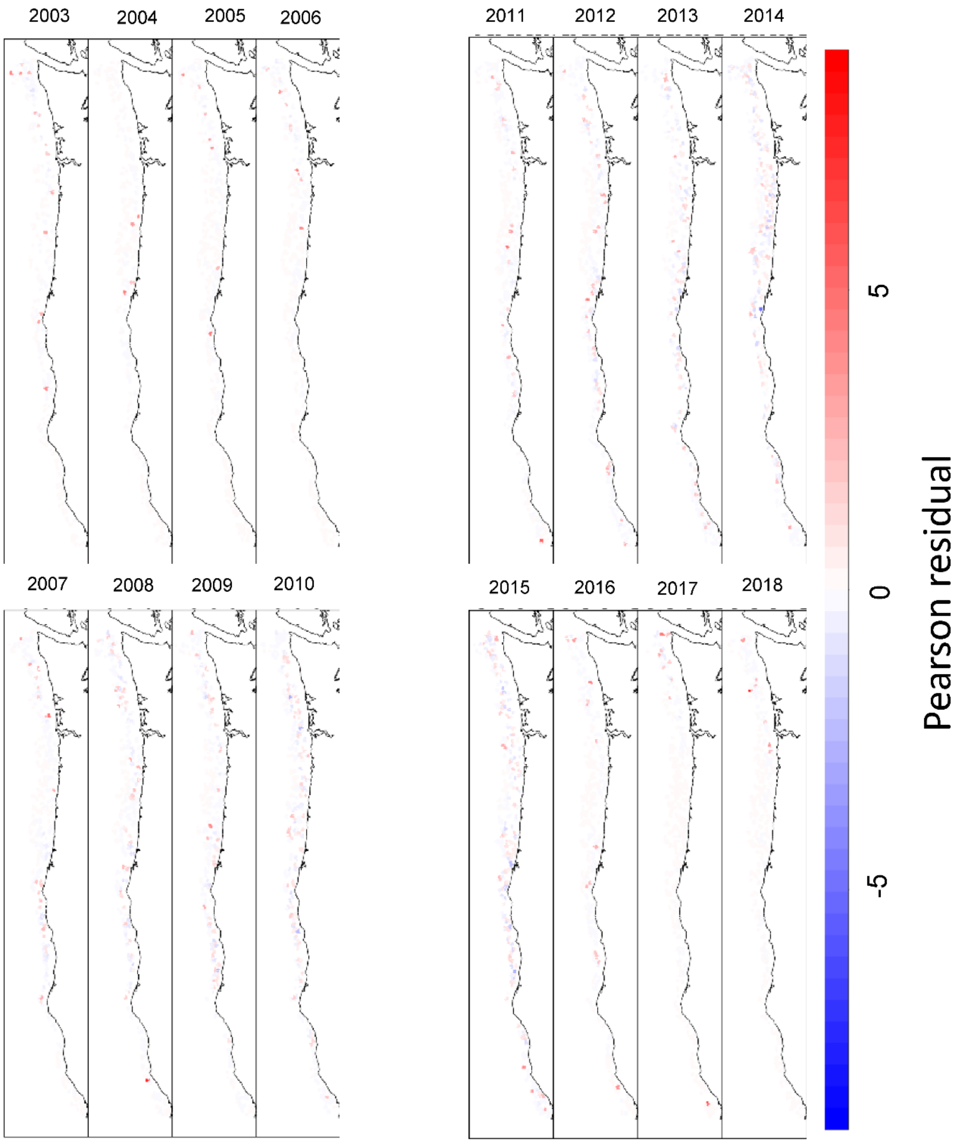

Fig CC. Spatial residuals for occurrence for arrowtooth flounder.

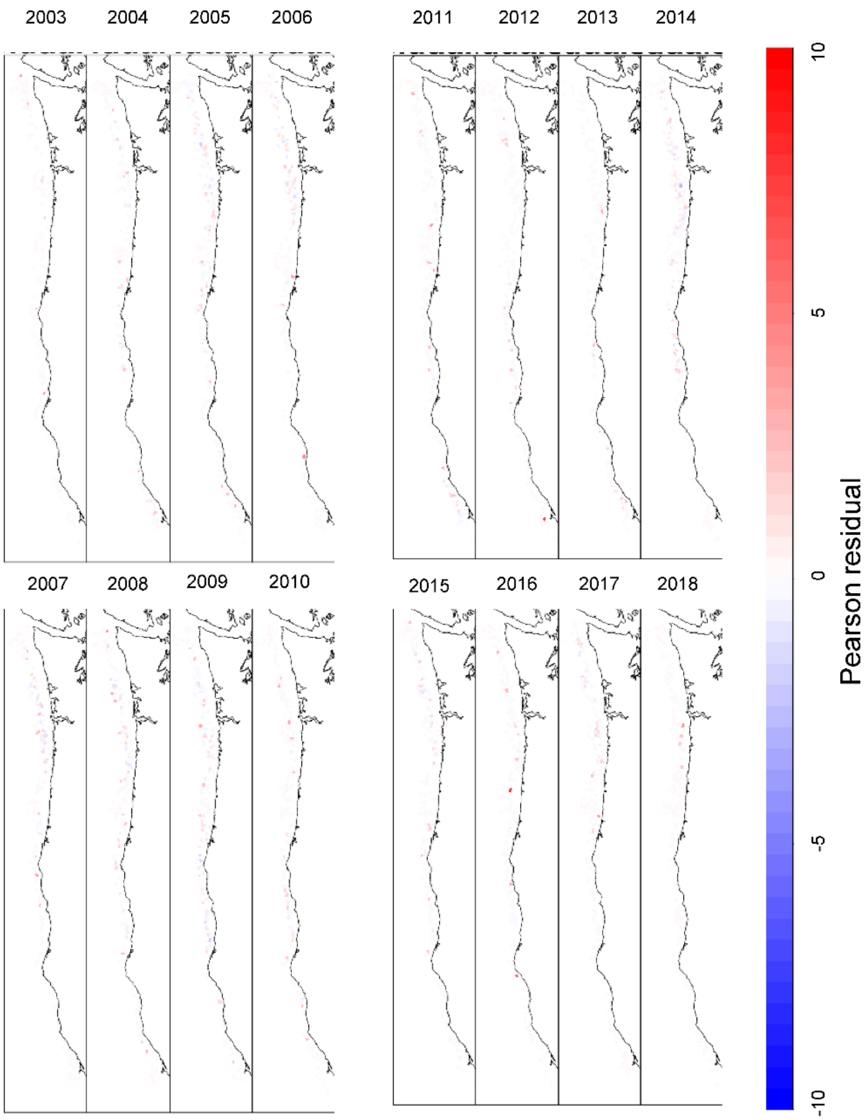

Fig DD. Spatial residuals for occurrence for darkblotched rockfish.

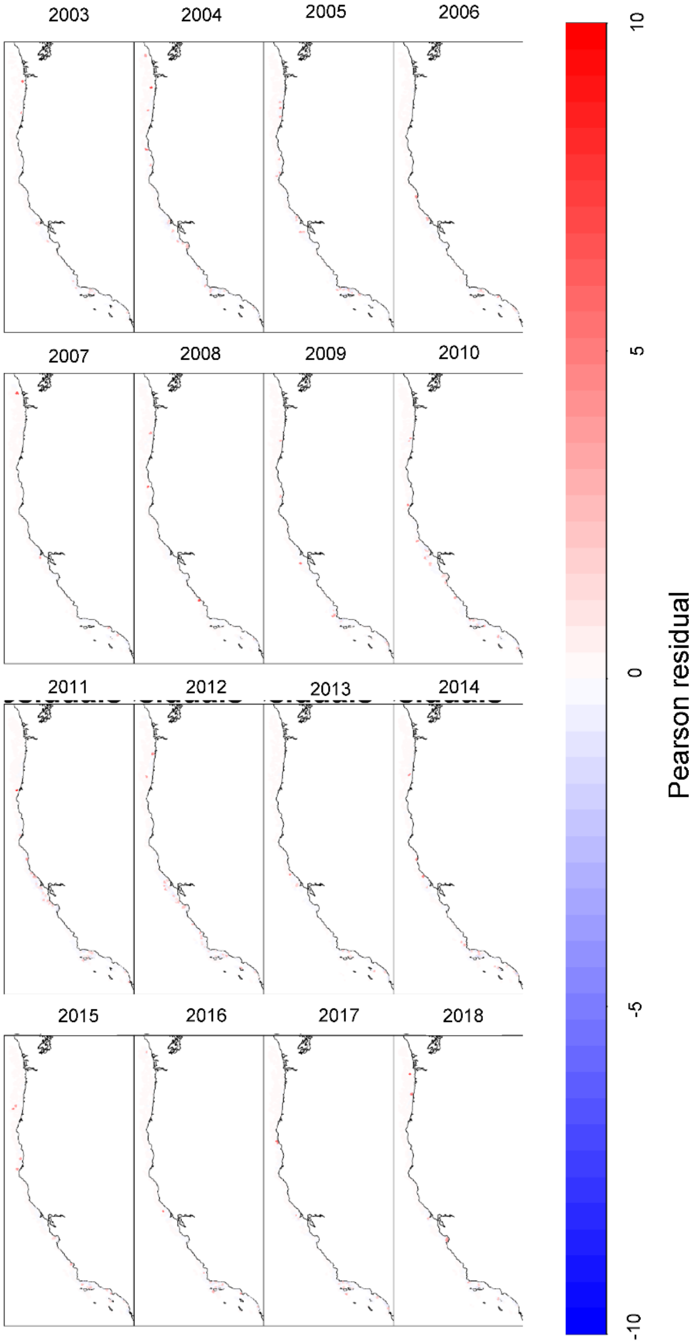

Fig EE. Spatial residuals for occurrence for Dover sole.

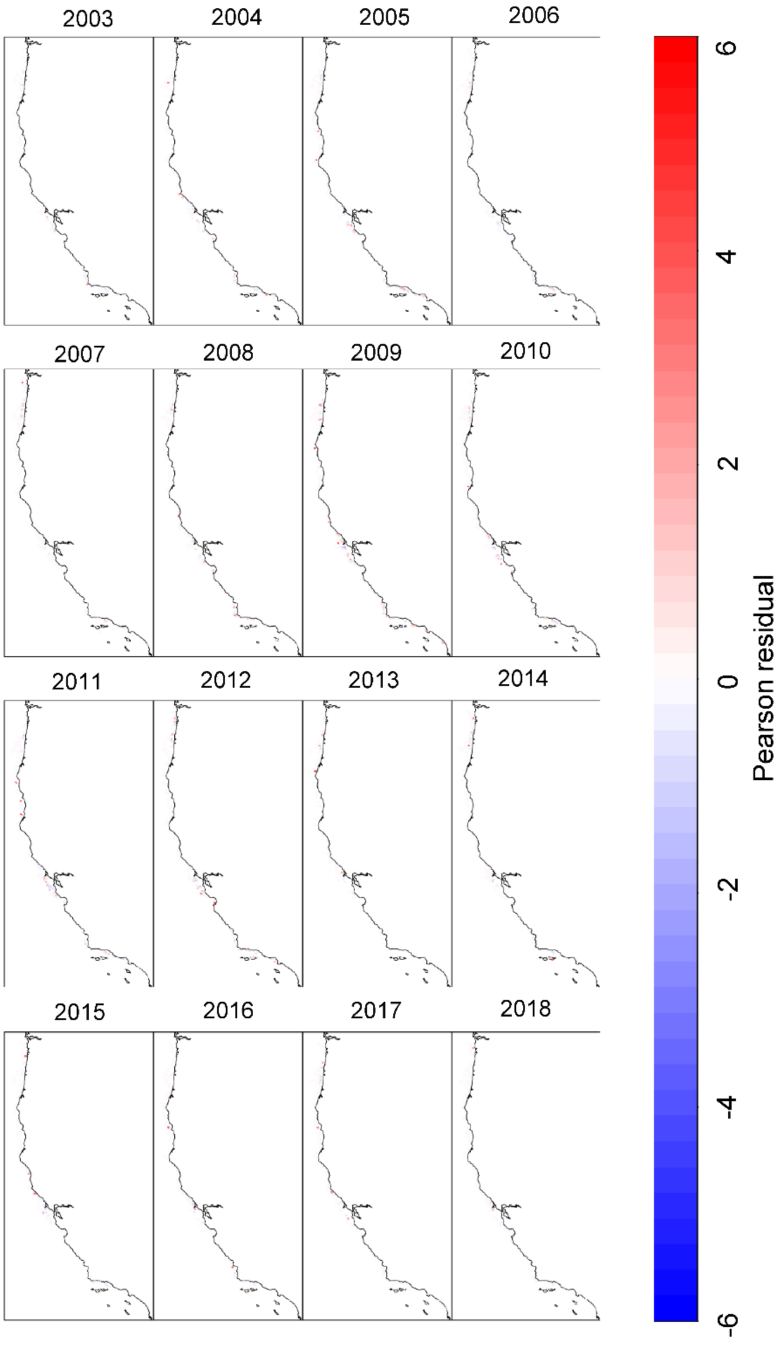

Fig FF. Spatial residuals for occurrence for English sole.

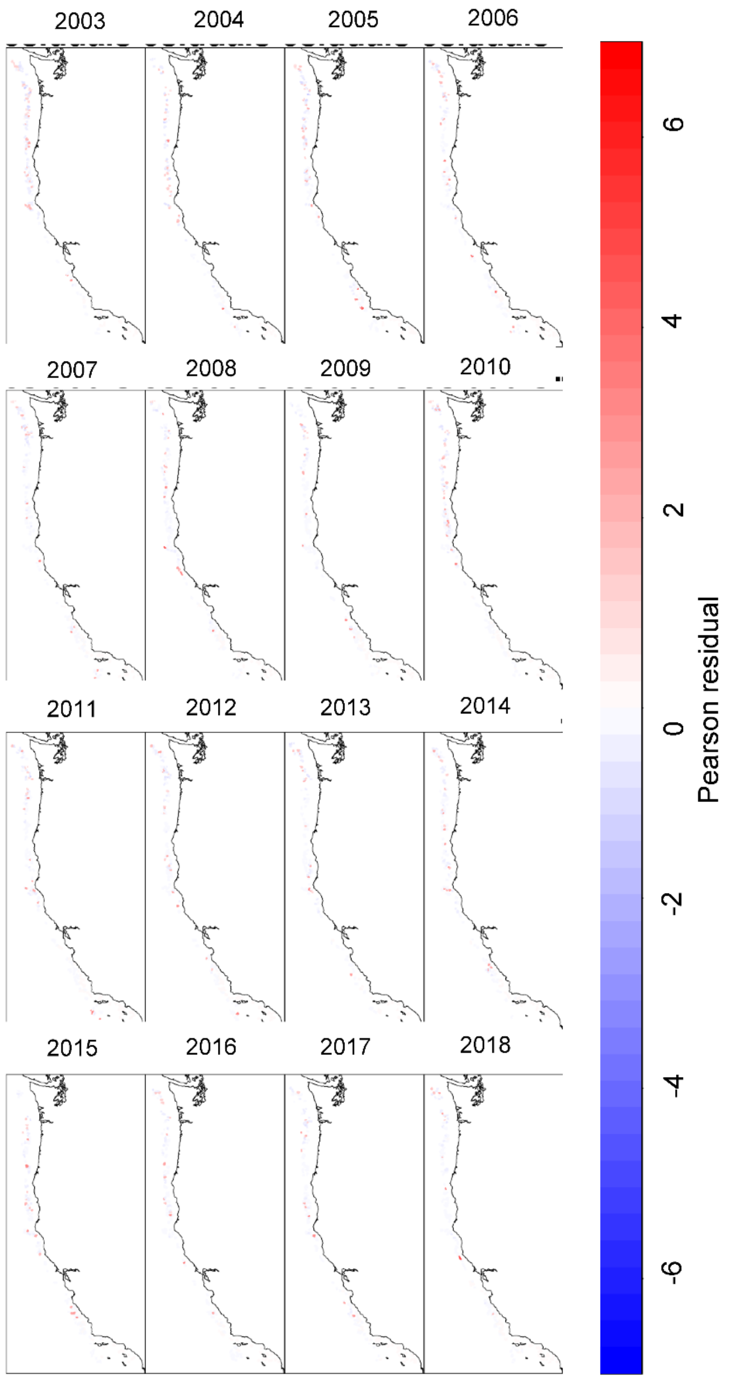

Fig GG. Spatial residuals for occurrence for Pacific greenling.

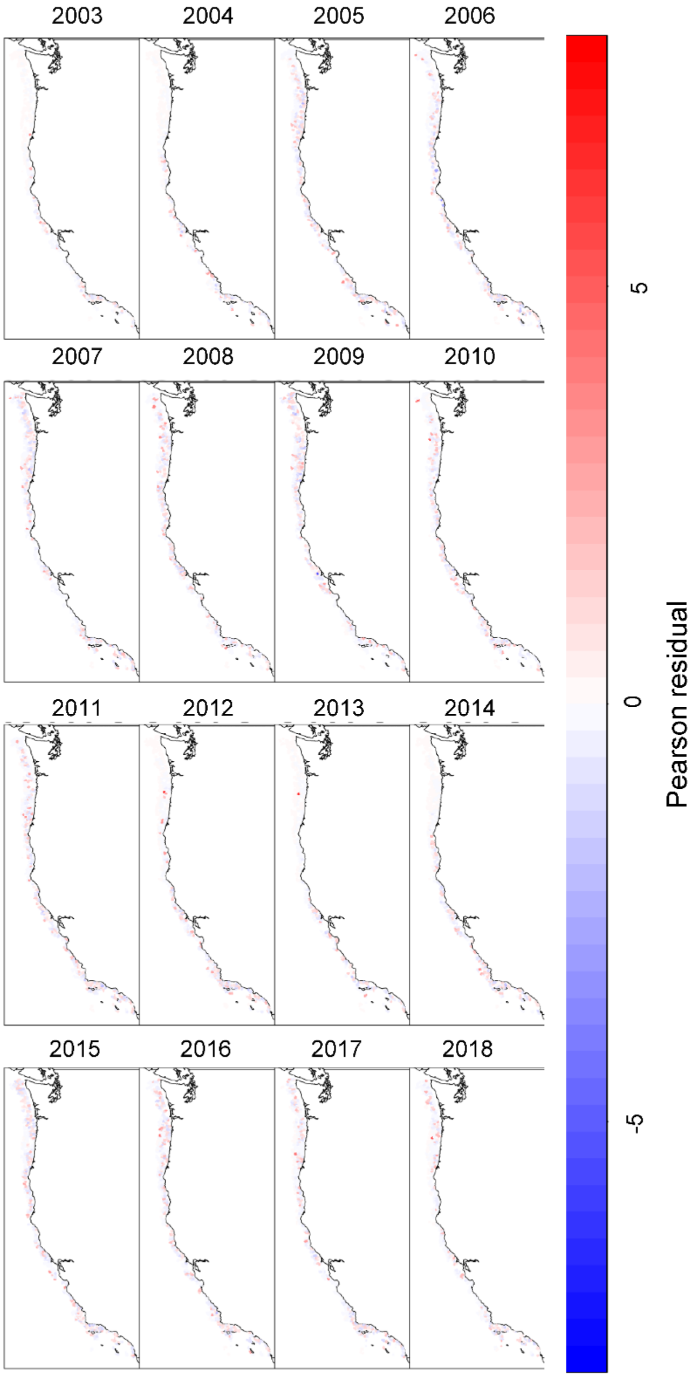

Fig HH. Spatial residuals for occurrence for Pacific hake.

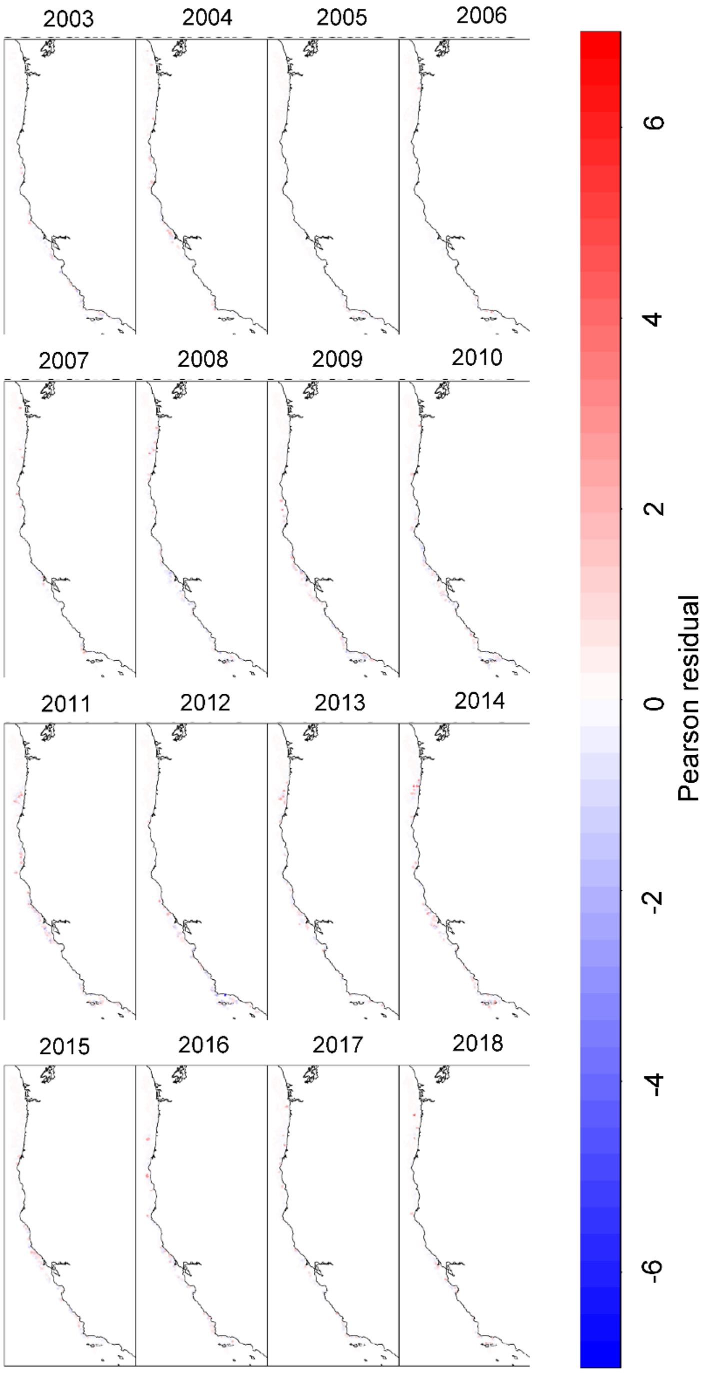

Fig II. Spatial residuals for occurrence for Lingcod.

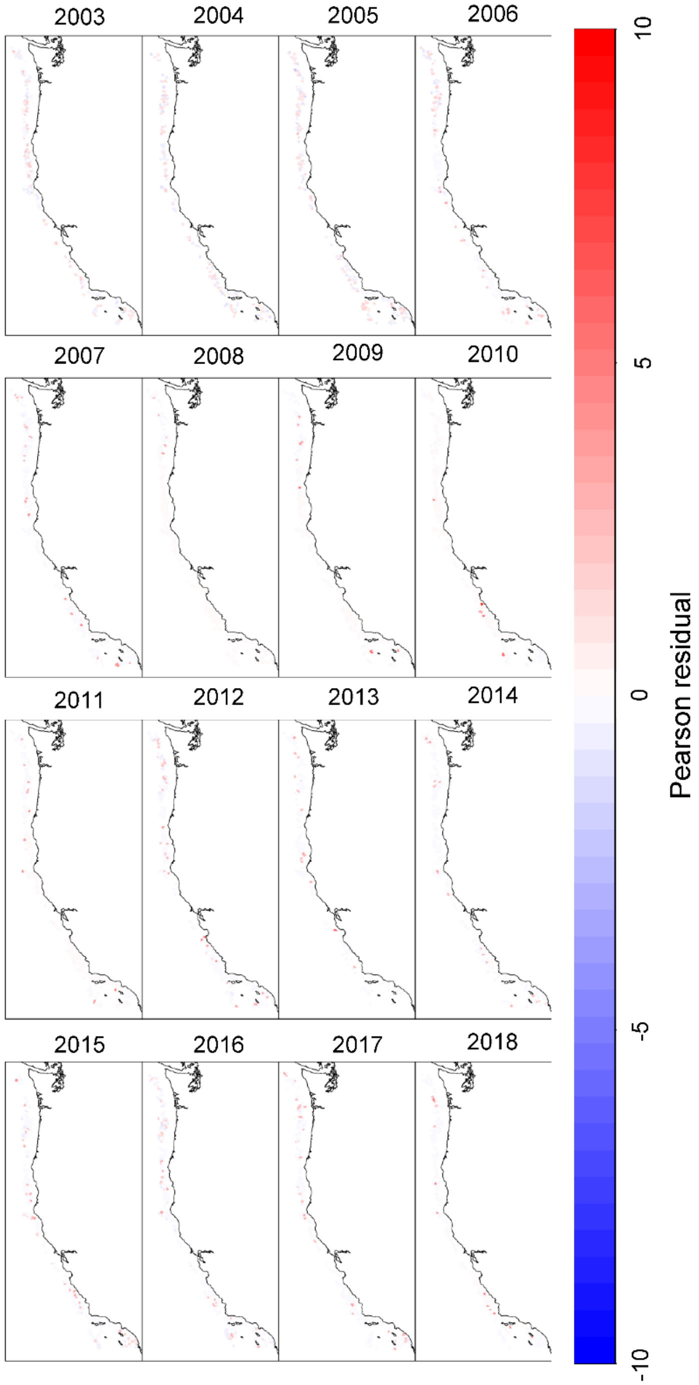

Fig JJ. Spatial residuals for occurrence for longspine thornyhead.

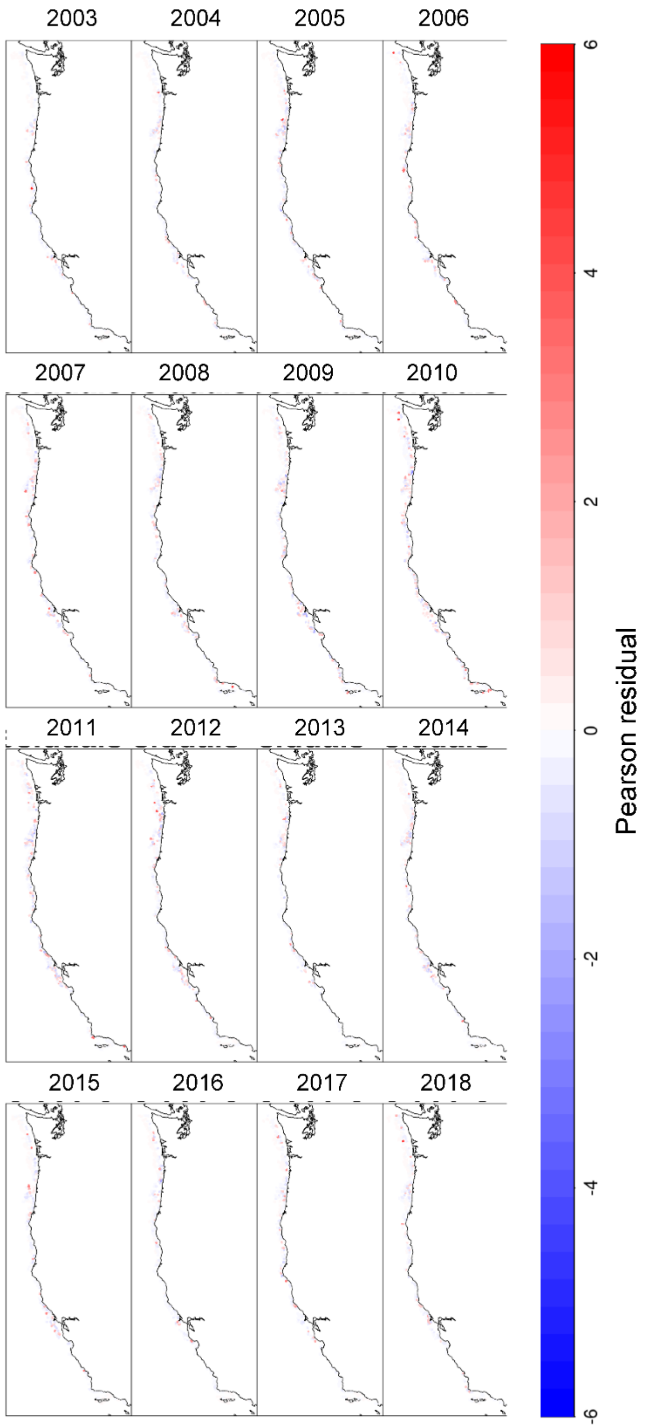

Fig KK. Spatial residuals for occurrence for Petrale sole.

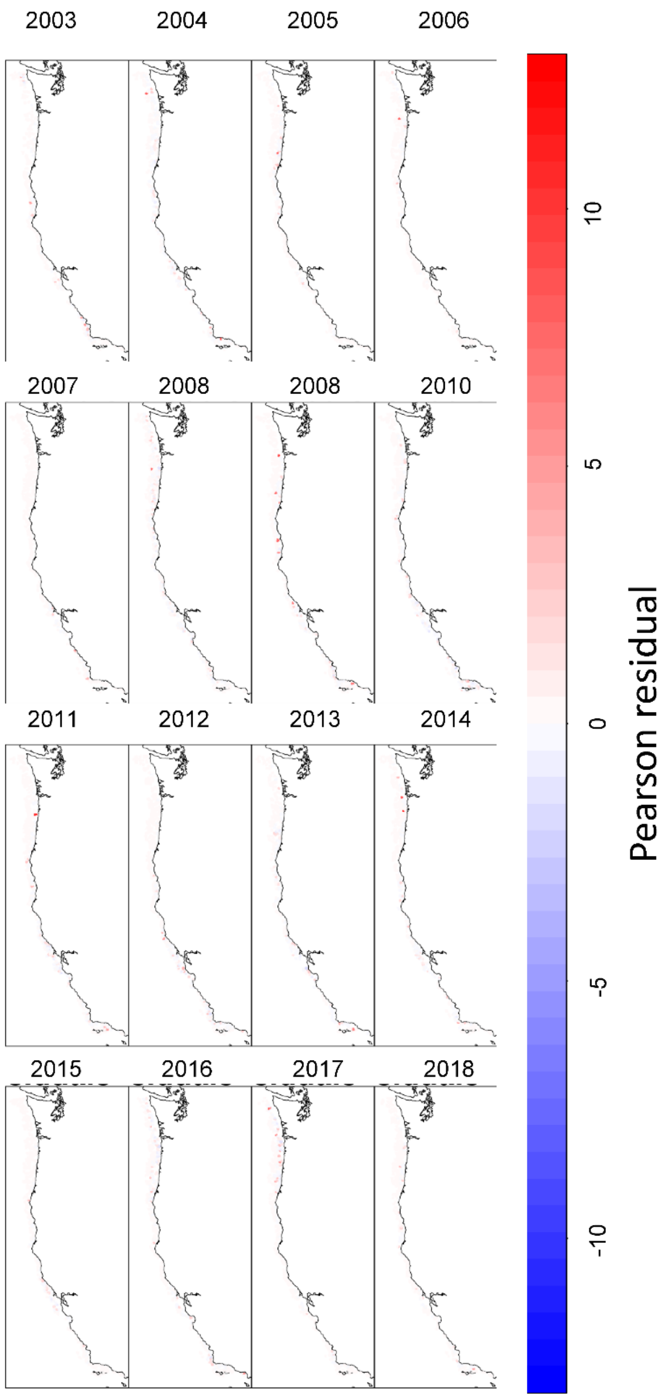

Fig LL. Spatial residuals for occurrence for sablefish.

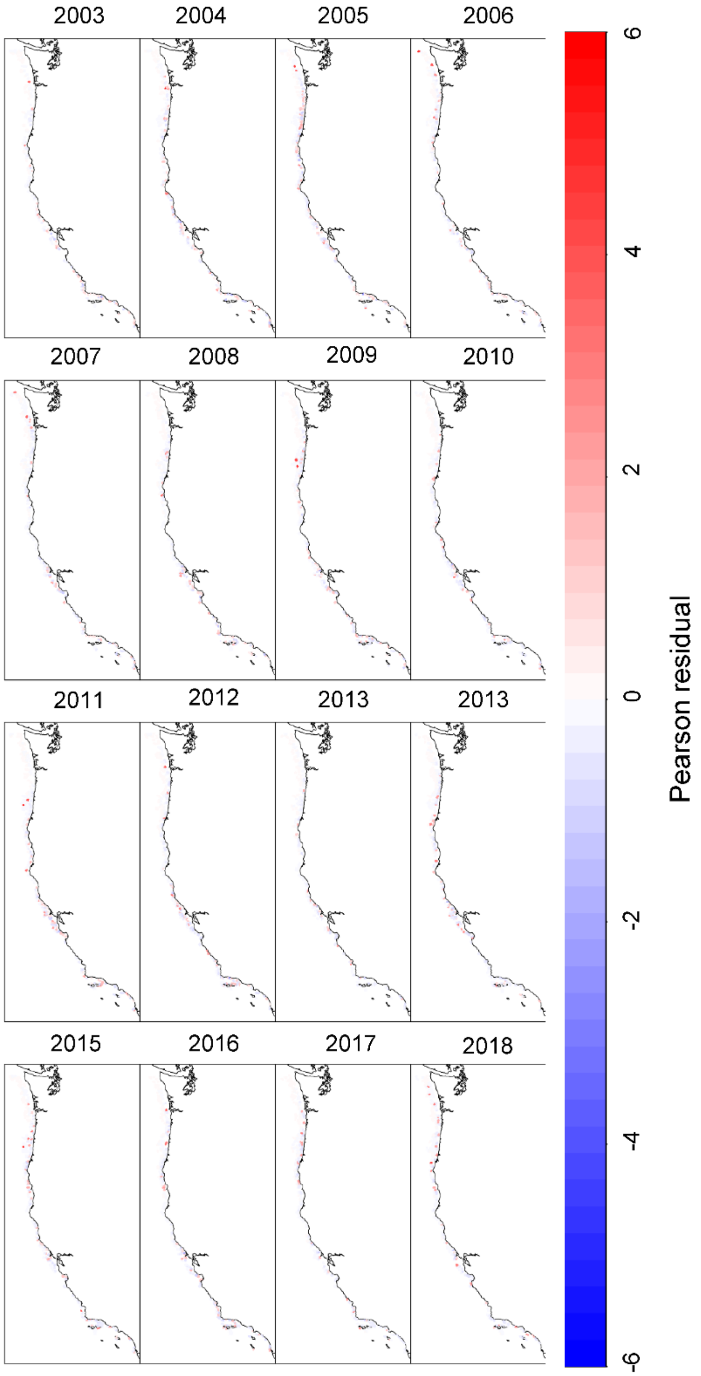

FigM M. Spatial residuals for occurrence for Pacific sanddab.

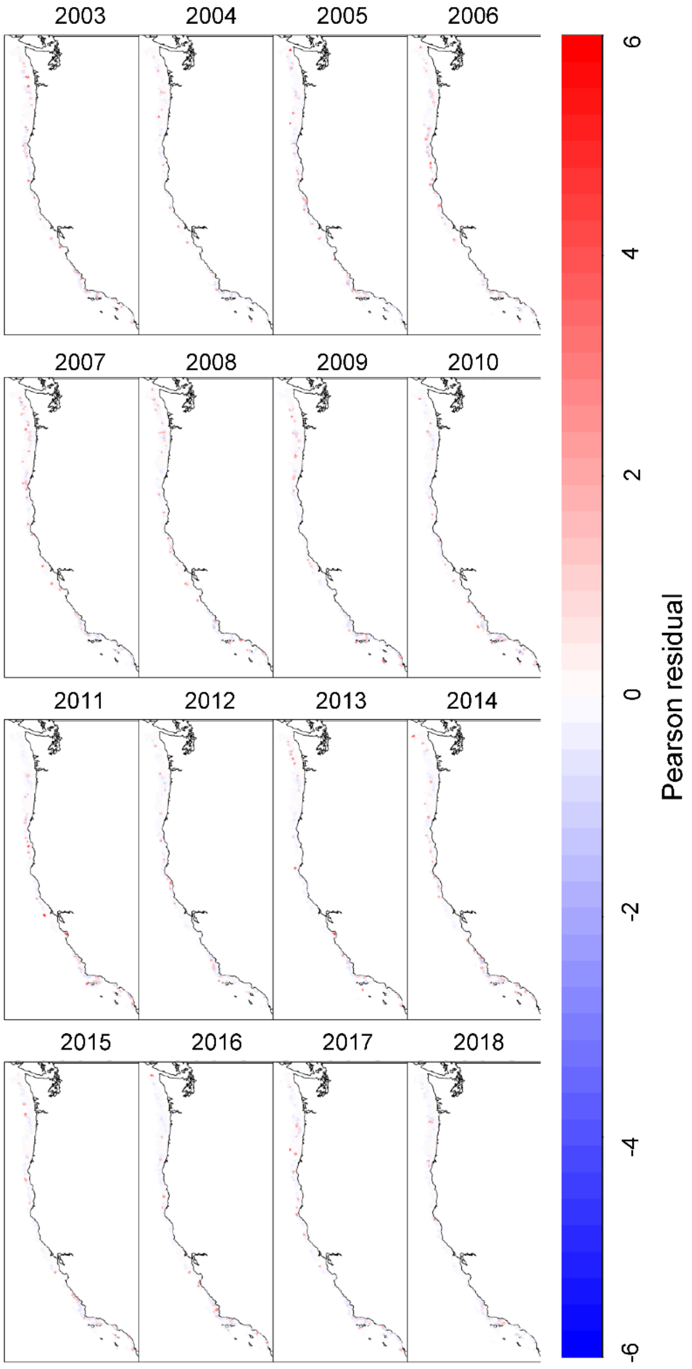

Fig NN. Spatial residuals for occurrence for sharpnose rockfish.

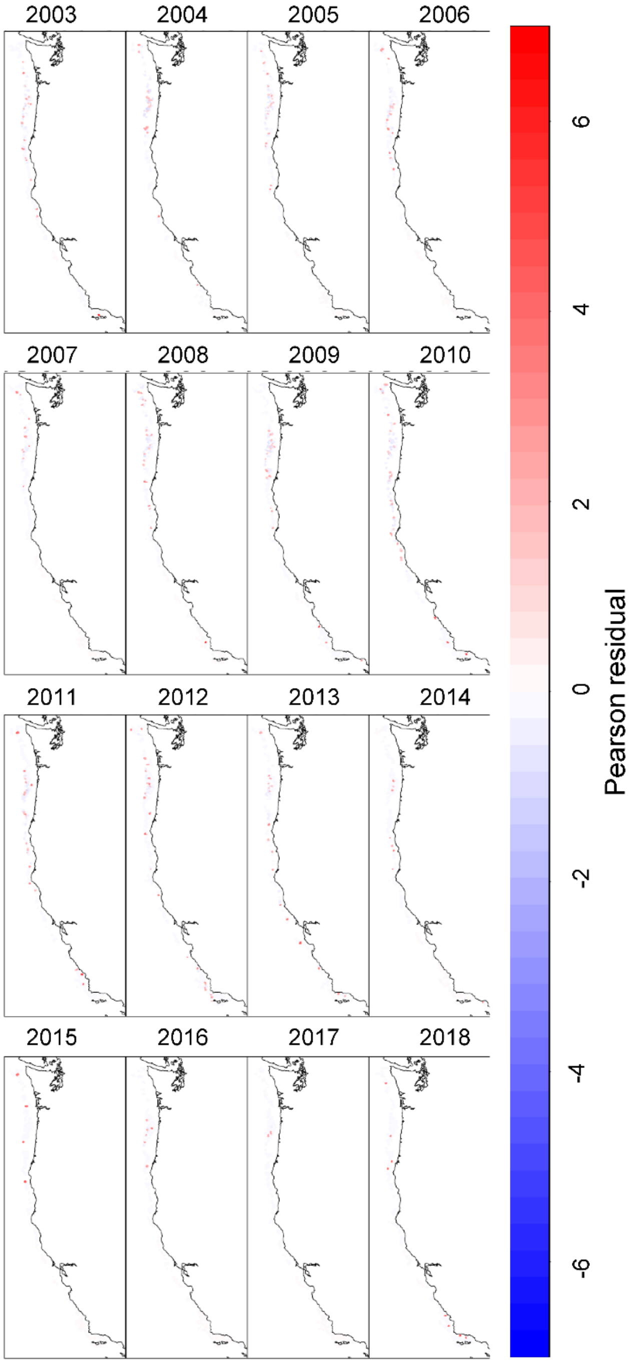

Fig O. Spatial residuals for occurrence for shortspine thornyhead.
